# Photoinduced Electron vs. Concerted Proton Electron Transfer Pathways in Sn^IV^ (l‐Tryptophanato)_2_ Porphyrin Conjugates

**DOI:** 10.1002/chem.202005487

**Published:** 2021-05-02

**Authors:** Mirco Natali, Agnese Amati, Nicola Demitri, Elisabetta Iengo

**Affiliations:** ^1^ Department of Chemical, Pharmaceutical and Agricultural Sciences (DOCPAS) University of Ferrara Via L. Borsari 46 44121 Ferrara Italy; ^2^ Centro Interuniversitario per la Conversione Chimica dell'Energia Solare (SolarChem) sez. di Ferrara, Via L. Borsari 46 44121 Ferrara Italy; ^3^ Department of Chemical and Pharmaceutical Sciences University of Trieste Via L. Giorgieri 1 34127 Trieste Italy; ^4^ Electra-Sincrotrone Trieste S.S. 14 Km 163.5 in Area Science Park 34149 Basovizza Trieste Italy; ^5^ Current address: Leiden Institute of Chemistry Leiden University Einsteinweg 55 2333CC Leiden The Netherlands

**Keywords:** electron transfer, H bond, proton coupled electron transfer, tin(IV) porphyrin, tryptophan

## Abstract

Aromatic amino acids such as l‐tyrosine and l‐tryptophan are deployed in natural systems to mediate electron transfer (ET) reactions. While tyrosine oxidation is always coupled to deprotonation (proton‐coupled electron‐transfer, PCET), both ET‐only and PCET pathways can occur in the case of the tryptophan residue. In the present work, two novel conjugates **1** and **2**, based on a Sn^IV^ tetraphenylporphyrin and Sn^IV^ octaethylporphyrin, respectively, as the chromophore/electron acceptor and l‐tryptophan as electron/proton donor, have been prepared and thoroughly characterized by a combination of different techniques including single crystal X‐ray analysis. The photophysical investigation of **1** and **2** in CH_2_Cl_2_ in the presence of pyrrolidine as a base shows that different quenching mechanisms are operating upon visible‐light excitation of the porphyrin component, namely photoinduced electron transfer and concerted proton electron transfer (CPET), depending on the chromophore identity and spin multiplicity of the excited state. The results are compared with those previously described for metal‐mediated analogues featuring Sn^IV^ porphyrin chromophores and l‐tyrosine as the redox active amino acid and well illustrate the peculiar role of l‐tryptophan with respect to PCET.

## Graphical abstract

**Photoinduced electron transfer** (ET) or concerted proton‐electron transfer (CPET) occur in tin(IV) porphyrin‐tryptophan conjugates in dichloromethane in the presence of an organic base depending on the chromophore identity and spin multiplicity of the excited state which point out how peculiar the l‐tryptophan amino acid is with respect to charge transfer reactions.

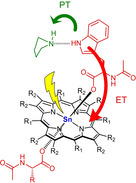

## Introduction

Tyrosine (TyrOH) and tryptophan (TrpNH) are key amino acids used in many biological systems to promote charge transfer processes.[^1^, ^2^] Within this context, the diverse redox and acid‐base properties of such amino acidic residues make their reactivity towards electron transfer (ET) substantially different. Due to the high oxidation potential of the phenol group (E° ∼ +1.5 V vs. NHE for the TyrOH/TyrOH^.+^ couple) and the low p*K*_a_ of the TyrOH^.+^ species (p*K*_a_=−2 in aqueous solution),^[3]^ tyrosine oxidation involves a proton‐coupled electron‐transfer (PCET) process, namely the coupling of a redox step (ET) with a proton transfer (PT) to an accepting base. On the other hand, in the case of tryptophan, the relatively low oxidation potential (E° ∼ +1.2 V vs. NHE for the TrpNH/TrpNH^.+^ couple) combined with the higher p*K*_a_ of the TrpNH^.+^ moiety (p*K*_a_=4.7 in aqueous solution)^[3]^ are such that, depending on the environmental conditions, tryptophan oxidation may actually involve either the TrpNH^.+^ radical cation (ET) or the TrpN^.^ neutral radical species (PCET).[^1^, ^3^] For example, in the photoactivation mechanism of *E. Coli* DNA photolyase,^[4]^ the first oxidation step promoted by the excited‐state of the flavin adenine dinucleotide follows an ET‐only mechanism with formation of a TrpNH^.+^ radical cation (W382^.+^), while the subsequent hole transfer process to a terminal tryptophan residue (W306) is accompanied by deprotonation yielding a neutral TrpN^.^ species (PCET). ET‐only processes are observed also in long‐range electron transfer within protein mutants mediated by tryptophan residues.^[5]^

PCET reactions involving redox active amino acids may occur either through stepwise ET‐PT/PT‐ET pathways or via a concerted mechanism (concerted proton electron transfer, CPET).[^2^, ^3^, ^6^] The latter implies the transfer of both electron and proton in a single, concerted kinetic event. CPET is usually favored on thermodynamic basis as it avoids the formation of high energy intermediates, while it may present kinetic barriers associated to the simultaneous involvement of both electron and proton motion.^[2]^

Many approaches have been adopted to study PCET reactions and understand the related mechanistic requirements. These includes electrochemical methods,[^7^, ^8^] the use of dark chemical oxidants[^9^, ^10^, ^11^] or photogenerated ones,[^12^, ^13^] as well as photoinduced PCET.[^14^, ^15^, ^16^, ^17^] While the behavior of tyrosine towards oxidation is rather well‐established from the large amounts of experimental data gathered, less is known in regard to tryptophan reactivity in the context of PCET. For instance, investigation of tryptophan oxidation in water using Os(bpy)_3_^3+^ (bpy=2,2’‐bipyridyne) as the chemical oxidant^[9]^ argued for a concerted proton‐electron transfer (CPET) mechanism. Similar conclusions were drawn in flash‐quench experiments on different covalently‐linked Ru(R_2_bpy)_3_^2+^‐tryptophan dyads (R_2_bpy=4,4’‐disubstituted‐2,2’‐bipyridyne),^[12]^ although this interpretation was subsequently questioned.^[18]^

In general, the peculiar redox and acid‐base properties of the tryptophan residue render the driving forces for ET and CPET comparable and the two processes can be in kinetic competition, with the ET process being typically more favored due to reduced mechanistic requirements (lower reorganization energy) with respect to the concerted pathway.^[1]^ Accordingly, the experimental conditions adopted (type of oxidant used, proton accepting base, etc.) can play a determining role in the type of oxidation mechanism.

**Scheme 1:**
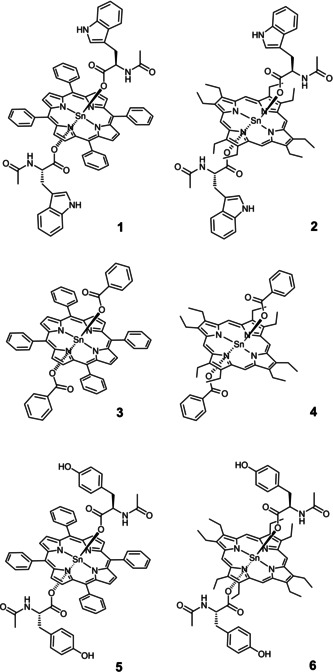
Molecular structures of novel conjugates **1**, **2**, model compounds **3**, **4**, and previously reported conjugates **5**, **6**.^[20]^

Within this framework, we have recently reported the photophysical investigation of two metal‐mediated conjugates consisting of a Sn^IV^‐porphyrin bearing two tyrosinato axial ligands (**5** and **6**, Scheme 1).^[20]^ For these systems excitation of the chromophore with visible light, in CH_2_Cl_2_ and in the presence of organic bases of suitable strength, triggered CPET with reduction of the porphyrin component, oxidation of the tyrosine residue, and concomitant proton transfer to the base. Importantly, CPET quenching rates and yields were strongly dependent on the porphyrin chromophore and on the base used, with pyrrolidine providing the largest quenching efficiency.^[20b]^ For these systems, diradical recombination was always faster than formation thus implying a photoacid behavior of the porphyrin‐tyrosine conjugates.^[20b]^ From these premises, we focused over possible progresses and improvements by variation of the redox active amino acid from tyrosine to tryptophan. Two new Sn^IV^(l‐tryptophanato)_2_‐porphyrin conjugates, differing for the substituents at the porphyrin periphery, alongside with the corresponding appropriate model compounds, i. e., featuring benzoate axial groups, commonly employed for comparative studies,[^20^, ^21^] were prepared, fully characterized, and studied (**1**–**4**, Scheme 1). The single crystal X‐ray analysis of **1**, while confirming the H‐donor character of the aa –NH indole side group, evidences some distinct peculiar features with respect to the parent Sn^IV^(l‐tyrosinato)_2_‐porphyrin conjugate (**5**), earlier reported.^[20a]^ A detailed photophysical investigation of **1**–**4** in CH_2_Cl_2_ in the presence of pyrrolidine was performed. The results show that different quenching pathways (photoinduced ET vs. CPET) can be selectively promoted upon visible light excitation depending on the Sn^IV^ porphyrin chromophore and on the spin multiplicity of the excited state.

## Results and Discussion

### Synthesis and characterization

Straightforward preparation of **1**–**4** as pure microcrystalline materials in high yields was done by the previous established procedure (see Experimental Section).[^20^, ^21^] A comprehensive and detailed characterization of **1** and **2** is reported in the Supporting Information (Figure S1–S10). Importantly, it comprises also 2D ^1^H‐^119^Sn heteronuclear correlation experiments that may serve to the scientific community addressing similar derivatives.

**Figure 1:**
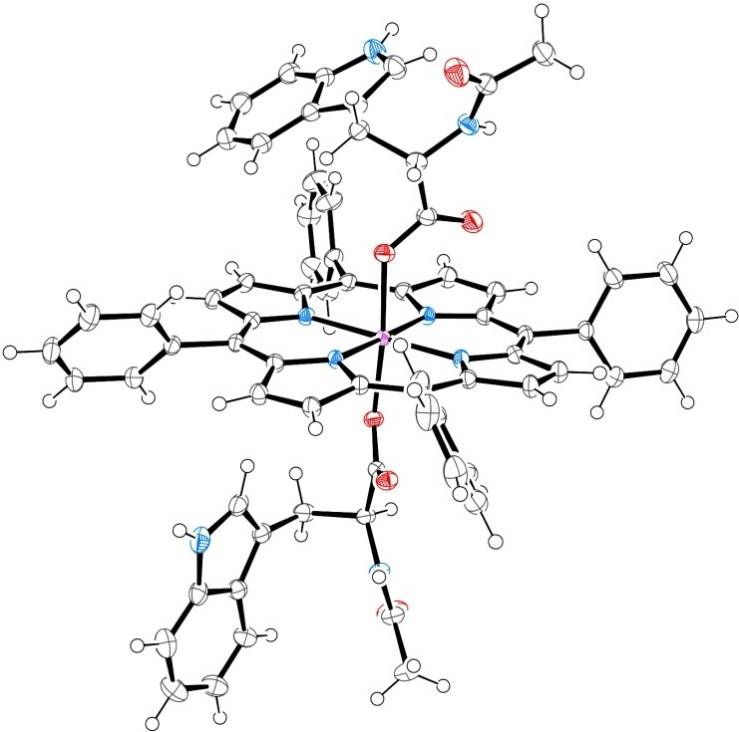
X‐ray structure of conjugate **1**, ellipsoids are drawn at 50 % probability. Solvent molecules are omitted for clarity; color code: oxygen, red; nitrogen, blue; tin, purple.

Crystals of conjugate **1**, suitable for X‐ray diffraction analysis, were obtained by slow vapor diffusion of *n*‐hexane over a concentrated solution in CHCl_3_. The resulting structure is reported in Figure 1 (see also Figures S11–S13 and Tables S1,S2 of the Supporting Information). **1** crystallizes in the chiral *P*2_1_ space group and the molecular model show the expected tryptophan Cα stereocenter configurations (*S*), further confirmed by the refined Flack parameter.^[22]^ The coordination sphere around the Sn^IV^ center presents bond lengths and angles in line with those already reported in similar systems (see also the comparison in Table S2).[^20a^, ^21c^, ^23^] Arrays of **1** develop along diagonal unit cells concatenated by hydrogen bonds involving the indole ‐NH and the acetylated carbonyl groups of equivalent tryptophan residues pertaining to adjacent unit cells (d_NH_ ⋅ ⋅ ⋅ _OC_=2.829(5) Å and d_NH_ ⋅ ⋅ ⋅ _OC_=2.862(5) Å, Figure S12A). The H‐donor NH group of the amidic terminal is instead involved in hydrogen bonds with chloroform solvent molecules (d_NH_ ⋅ ⋅ ⋅ _Cl_=3.68(1) Å). The monoclinic crystal form of **1** shows CH ⋅ ⋅ ⋅ π interactions between peripheral phenyl protons and indole rings of neighboring porphyrin and amino acid residues, respectively (intramolecular d_CH_ ⋅ ⋅ ⋅ _π_=3.510(4)Å ‐ intermolecular d_CH_ ⋅ ⋅ ⋅ _π_=3.641(5)Å, Figure S12B). These intra‐ and inter‐molecular interactions efficiently stabilize a bent conformation of the amino acid sidechain on only one side of the porphyrin macrocycle, resulting in the two tryptophan residues coordinated to the same tin center to adopt significantly different indole‐ring orientations (Figure S13). This difference is peculiar and has not been observed for adduct **5** ^[20a]^ and analog systems^[23]^ and may be related to packing effects.

The crystal structure of model compound **4** has been also resolved by X‐ray diffraction analysis and is reported in Figure S14. Additional details can be found in the Supporting Information (Table S1, S2 and Figure S15, S16).

### Photophysical behavior in CH_2_Cl_2_

**Figure 2:**
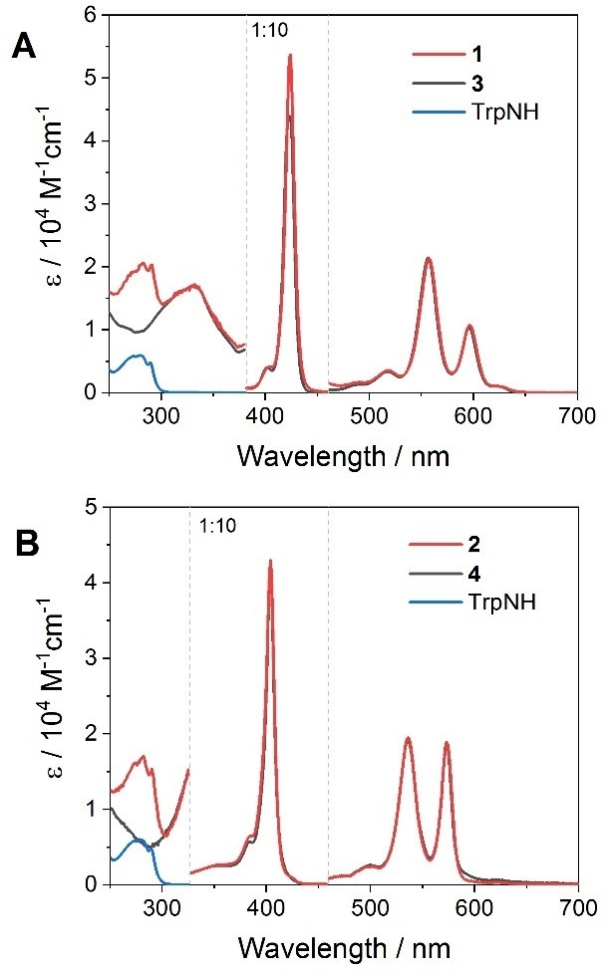
Comparison of the absorption spectra of conjugates and model compounds in CH_2_Cl_2_: A) **1**, **3**, and *N‐*acetyl‐l‐tryptophan; B) **2**, **4**, and *N‐*acetyl‐l‐tryptophan.

The absorption spectra of conjugate **1** and **2** in CH_2_Cl_2_ (Figure 2) display the typical absorption features of metallo‐porphyrins of the regular type,^[24]^ namely two Q‐bands in the 500–600 nm range (maxima at 518, 557, 596 nm and 499, 536, 573 nm for **1** and **2**, respectively) and a more intense Soret‐band at around 400–450 nm (maxima at 422 nm and 404 nm for **1** and **2**, respectively). The structured band (with maxima at 274, 281, and 290 nm) of the tryptophan amino acid can be distinguished in the UV region of the spectrum. Thus, visible light excitation of both conjugates selectively promotes formation of the singlet excited state of the porphyrin unit. Importantly, the absorption spectra of both conjugates result as a perfect superposition of those of the corresponding model compounds (**3**, **4**) and that of *N*‐acetyl‐l‐tryptophan. Furthermore, the electrochemical potentials of **1** and **2**, obtained by cyclic voltammetry (CV), are comparable to those of their respective model compounds (Table 1). As for related metal‐mediated conjugates,[^20^, ^21^, ^25^, ^26^] these observations are consistent with the absence of relevant ground‐state interactions between the porphyrins and the tryptophan residues in **1** and **2**. This allows us to confidently use compounds **3** and **4** (for **1** and **2**, respectively) and *N‐*acetyl‐l‐tryptophan as reliable comparative models to assess the relevant energetics of the conjugates. A closer inspection of the CV data in Table 1 shows that in the case of **1** a charge transfer state of the type SnTPP^.−^‐TrpNH^.+^ may be in principle populated from the singlet excited state of the chromophore (ΔG°=−0.01 eV, considering an E^0–0^=2.10 eV and neglecting electrostatic work terms), whereas in the case of **2** the SnOEP^.−^‐TrpNH^.+^ state is largely up‐hill with respect to the singlet excited level of the SnOEP component (ΔG°=+0.24 eV, considering an E^0–0^=2.16 eV and neglecting electrostatic work terms). These results are relevant to the photophysics of the porphyrin‐tryptophan adducts as is shown hereafter.

**Table 1:** Electrochemical data of conjugates and model compounds.^[a]^

	*E*_ox_ (V)	*E*_red_ (V)
**1**	+0.73 ^[b]^	−1.37
**2**	+0.72 ^[b]^	−1.61
**3**	+0.91	−1.37
**4**	+0.79	−1.68
*N*‐acetyl‐l‐tryptophan	+0.72^[b]^	/

[a] Obtained by cyclic voltammetry (CV) in N_2_‐purged CH_2_Cl_2_ (0.1 M TBAPF6) at 298 K, scan rate 100 mV/s, using GC as WE, Pt as CE, and SCE as reference, potentials are referred to Fc/Fc^+^ used as an internal standard (Figure S17–S20); [b] irreversible wave, peak potential given.

**Figure 3:**
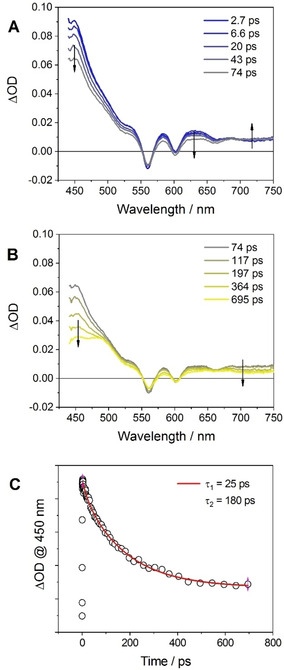
Ultrafast spectroscopy (excitation at 400 nm) of conjugate **1** in CH_2_Cl_2_: spectral evolution between A) 2.7–74 ps and B) 74–695 ps, C) kinetic analysis at 450 nm.

The fluorescence of the Sn^IV^ porphyrin in **1** in CH_2_Cl_2_ (maxima at 604 and 658 nm) is quenched by ca. 75 % when compared to that of an optically‐matched solution of model compound **3** (Figure S21). Also, a comparable decrease of the singlet excited state lifetime is observed by TC‐SPC (0.31 and 1.15 ns for **1** and **3**, respectively). This trend is consistent with the occurrence of a photoinduced ET process with formation of a SnTPP^.−^‐TrpNH^.+^ charge transfer state, as postulated from purely thermodynamic considerations (see above). Ultrafast spectroscopy was performed to better analyze the singlet quenching process in **1**. The prompt spectrum (time delay of 2.7 ps, Figure 3A) is characterized by a positive absorption with maximum at 450 nm and a tail at longer wavelengths with superimposed Q‐band bleaching and stimulated emission (relative minima at 560, 602, and 660 nm). This latter corresponds to the differential spectrum of the singlet excited state of the SnTPP unit.^[21e]^ The subsequent spectral evolution (Figure 3A,B) shows a biphasic behavior with a fast component (t<70 ps) and a slower one (t>70 ps). The first process (Figure 3A) features a slight decrease of the transient absorption signal below 550 nm and between 570 and 670 nm concomitant to a slight growth of an absorption pattern above 700 nm, characteristic of the porphyrin radical anion.[^27^, ^28^] The second process (Figure 3B) is characterized by the decrease of the absorption at 450 nm with formation of a new maximum at 480 nm, while at longer wavelengths a decrease and flattening of the transient signal is observed. Interestingly, the final spectrum matches the transient signature of the ^3^*SnTPP triplet excited state (Figure S22).^[20]^ Kinetic analysis (Figure 3C) yields time constants of τ_1_=25 ps and τ_2_=180 ps for the first and second process, respectively. The first one can be assigned to the formation of the SnTPP^.−^‐TrpNH^.+^ state from the singlet excited state of the SnTPP. Lack in the observation of substantial absorption from the porphyrin radical anion above 700 nm[^27^, ^28^] very likely suggests that, due to the very small energy gap between the ^1^*SnTPP and the SnTPP^.−^‐TrpNH^.+^ state, population of the charge transfer state (τ_1_=25 ps) is not quantitative and an equilibrium is established with predominant population of the SnTPP singlet.^[21a]^ The second process (τ_2_=180 ps) can be then assigned to the formation of the SnTPP triplet from the equilibrium admixture in competition with ground state decay.^[21a]^ The corresponding kinetics is indeed consistent with the singlet excited state lifetime measured by TC‐SPC (0.31 ns). The triplet excited state of the SnTPP in conjugate **1** is formed with 46 % efficiency upon photoexcitation of the porphyrin unit, as estimated by laser flash photolysis (Figure S22), and undergoes ground‐state decay with an average lifetime of τ≈160 μs (Figure S23). This clearly confirms that the triplet excited state of the SnTPP component in conjugate **1** is not quenched, as expected based on purely thermodynamic considerations. The photophysical behavior of conjugate **1** in CH_2_Cl_2_ is summarized in Figure S24.

The fluorescence of the SnOEP component in conjugate **2** in CH_2_Cl_2_ (maxima at 577 and 630 nm) has a comparable intensity with respect to that of model compound **4** (Figure S25). Consistently, similar singlet lifetimes have been measured by TC‐SPC (τ=0.88 and 0.90 ns for **2** and **4**, respectively). This result confirms that the singlet excited state of the Sn^IV^ porphyrin unit in **2** is not quenched by the pendant tryptophan residues. Also, the triplet excited state of the SnOEP component is not quenched in conjugate **2**. An average lifetime of τ≈70 μs can be estimated (Figure S26).[^29^, ^30^] Thus, the photophysical behavior of conjugate **2** clearly resembles that of model compound **4** (see the energy level diagram in Figure S29). This is indeed expected considering the more negative reduction potential of the SnOEP porphyrin component with respect to the SnTPP analogue (Table 1) that lifts the charge transfer state in **2** at a higher energy than in **1**.

### Photophysical behavior in CH_2_Cl_2_ with pyrrolidine

The photophysical behavior of both conjugates **1** and **2** has been then assessed in the presence of pyrrolidine as a base. As observed in both **5** and **6** as well as in related chromophore‐phenol dyads,[^20^, ^31^] the addition of pyrrolidine is expected to introduce a new deactivation channel of PCET nature yielding radical pair states of the type SnTPP^.−^‐TrpN^.^ ⋅ ⋅ ⋅ ^+^HPyr and SnOEP^.−^‐TrpN^.^ ⋅ ⋅ ⋅ ^+^HPyr for **1** and **2**, respectively. These processes are indeed expected to be favored on a thermodynamic standpoint upon excitation of the porphyrin chromophore in both conjugates. As a matter of fact, such PCET states should lie at lower energy than the corresponding ET‐only states (namely SnTPP^.−^‐TrpNH^.+^ and SnOEP^.−^‐TrpNH^.+^ for **1** and **2**, respectively) by a factor of 0.7 (±0.2) eV. This quantity (ΔG_PT_) corresponds to the driving force involved in the deprotonation of the oxidized tryptophan (TrpNH^.+^) by the pyrrolidine base, as possibly estimated from available acid‐base data.[^32^, ^33^, ^34^]

Furthermore, as a fundamental requirement to promote efficient CPET, pyrrolidine was shown to be involved in hydrogen‐bonding interactions with the indole NH group with an association constant of *K*_A_=5.3(±0.7) M^−1^ in CH_2_Cl_2_, as determined from a spectrophotometric titration experiment on model compound *N‐*acetyl‐l‐tryptophan using two different methodologies (Figure S30–S32).[^35^, ^36^] This value is lower than that measured for the hydrogen‐bonding between the same base and the hydroxide group of a tyrosine amino acid,^[20b]^ consistent with the lower donor ability of the NH vs. the OH group with respect to H‐bond.^[37]^

**Figure 4:**
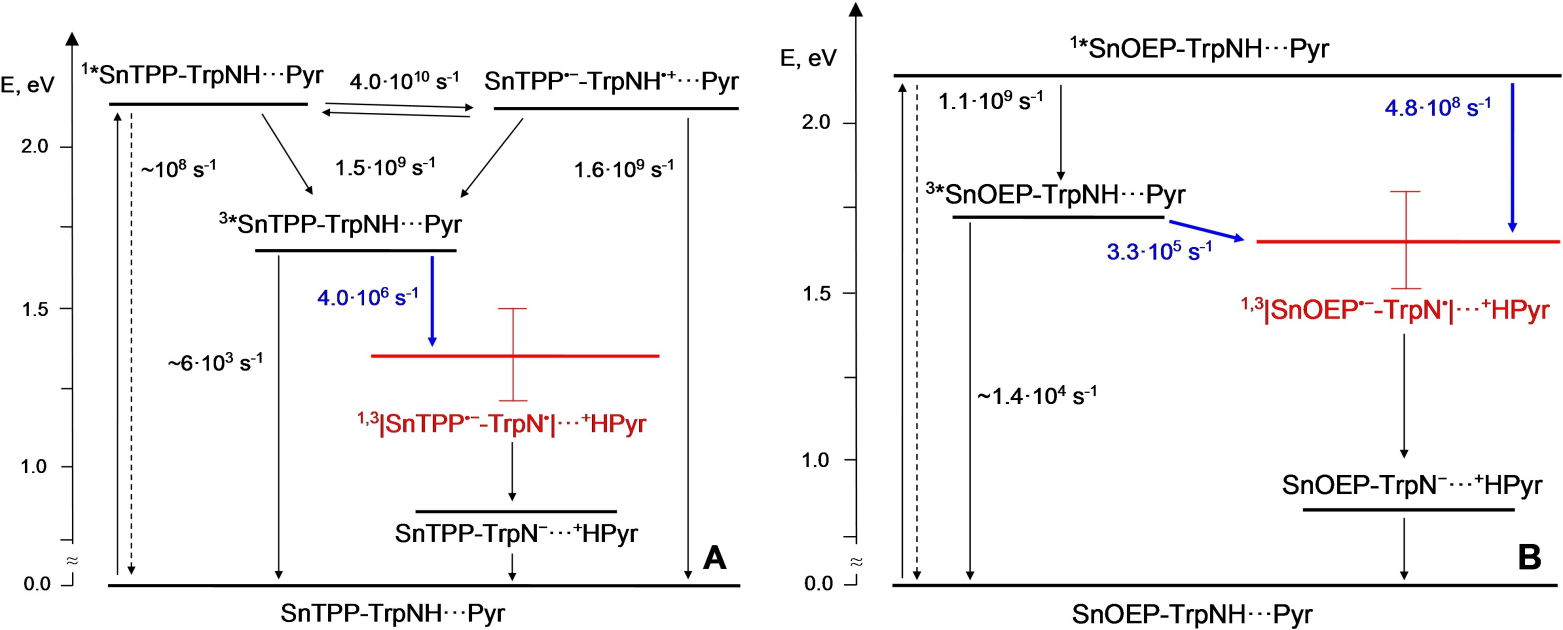
Energy level diagram of A) conjugate **1** and B) **2** with related processes and rates (the CPET quenching processes have been highlighted in blue, the corresponding rates represent the *k*_CPET_ determined from [Eq. (1)]). The energy of the singlet excited states was taken from the intersection of the normalized absorption/emission spectra, the energy of the triplet excited states from phosphorescence data,^[24]^ the energy of the radical pair states (in red) and the PT‐only states was derived from the combination of electrochemical and acid‐base data (see Supporting Information for further details). For sake of simplicity, the same energy was taken for the singlet and triplet diradicals.

The energy level diagrams of both conjugates **1** and **2** in CH_2_Cl_2_ in the presence of pyrrolidine, obtained from a combination of redox (Table 1) and protonation data (see Supporting Information for further details), are reported in Figure 4. These diagrams will be useful to assist the reader during the following discussion.

**Figure 5:**
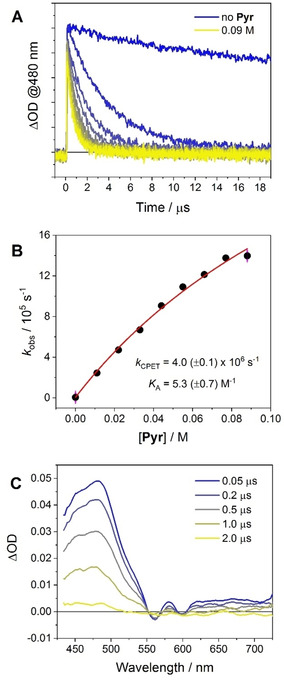
A) Relevant kinetic traces at 480 nm obtained by laser flash photolysis (excitation at 532 nm, 2 mJ) of **1** in N_2_‐purged CH_2_Cl_2_ in the presence of 0–0.09 M pyrrolidine; B) plot of the triplet decay rate *k* (obtained from a single exponential fitting of the decays in Fig. 3A) vs. pyrrolidine concentration and fitting of the experimental data according to [Eq. (1)]; C) transient absorption spectra obtained by laser flash photolysis (excitation at 532 nm, 2 mJ) of **1** in N_2_‐purged CH_2_Cl_2_ in the presence of 0.09 M pyrrolidine.

The fate of the singlet excited state of the porphyrin in **1** remains practically unaltered in the presence of pyrrolidine. As a matter of fact, comparable fluorescence intensities (between 25–32 % with respect to the emission of model compound **2**) and lifetimes (between 0.31–0.35 ns) have been recorded in CH_2_Cl_2_ upon subsequent additions of pyrrolidine up to 0.064 M (Figure S33). This clearly suggests that, even in the presence of a new, competitive deactivation pathway (i. e., photoinduced CPET), photoinduced ET is the dominating mechanism that deactivates the singlet excited state of SnTPP in conjugate **1**. On the other hand, the triplet excited state of SnTPP (populated with ca. 50 % efficiency upon photoexcitation, see above) is appreciably affected by the presence of the base. Increasing additions of pyrrolidine in the range 0–0.088 M cause indeed a faster deactivation of the triplet excited state, as monitored in the transient absorption maximum at 480 nm (Figure 5A). The quenching efficiency is dependent on the amount of pyrrolidine added, reaching an apparent saturation at high concentrations. Since direct, bimolecular ET quenching of ^3^*SnTPP by pyrrolidine can be excluded (second‐order rate constant of *k*_Q_=9.2×10^5^ M^−1^s^−1^, as estimated from a Stern‐Volmer analysis,^[20]^ which implies lower quenching yields at the pyrrolidine concentrations used), these observations point towards the occurrence of a CPET at the triplet excited state level involving formation of a ^3^|SnTPP^.−^‐TrpN^.^| ⋅ ⋅ ⋅ ^+^HPyr diradical (Figure 4A). A kinetic isotope effect (KIE) of 1.4(±0.1) was found comparing the triplet excited state decays of **1** in CH_2_Cl_2_ with pyrrolidine in the presence of 1 % v/v of either CH_3_OH or CD_3_OD (Figure S34), implying that H/D transfer is directly involved in the quenching process.^[38]^ This value is consistent with the one found for the parent conjugate **5** and other related systems.[^20b^, ^30^] Furthermore, application of the kinetic treatment used to account for CPET reactions ([Eq. (1)])[^20^, ^30^] which considers the pre‐association (with equilibrium constant *K*_A_) between the proton donating group (TrpNH in the present case) and the proton accepting base (pyrrolidine), turns out to be effective in modelling the observed dependence of the ^3^*SnTPP decay rates vs. pyrrolidine concentration.

(1) $$k_{obs}=k_{0}+k_{Q}\left[Pyr\right]+k_{CPET}\frac{K_{A}\left[Pyr\right]}{1+K_{A}\left[Pyr\right]}$$

Fitting of the experimental data according to [Eq. (1)], using a *k*_0_=6.2×10^3^ s^−1^ (from the average lifetime of τ=160 μs), a *k*_Q_=9.2×10^5^ M^−1^s^−1^,^[20]^ and the *K*_A_=5.3(±0.7) M^−1^ previously estimated (Figure 5B), yields a rate constant of *k*_CPET_=4.0(±0.1)×10^6^ s^−1^. The spectral evolution of the transient absorption of **1** in CH_2_Cl_2_ with 0.088 M pyrrolidine has been then monitored (Figure 5C). The prompt spectrum, measured at 50 ns time‐delay, corresponds to the transient signal of the SnTPP triplet.[^21a^, ^21e^] This spectrum subsequently decays to the baseline within a few μs without formation of new transient signatures (e. g., those expected for a radical pair state of the type ^3^|SnTPP^.−^‐TrpN^.^| ⋅ ⋅ ⋅ ^+^HPyr).[^27^, ^28^] This clearly means that the forward CPET quenching to yield the triplet diradical is slower than the corresponding recombination so that negligible accumulation of such a radical pair can be attained.

**Figure 6:**
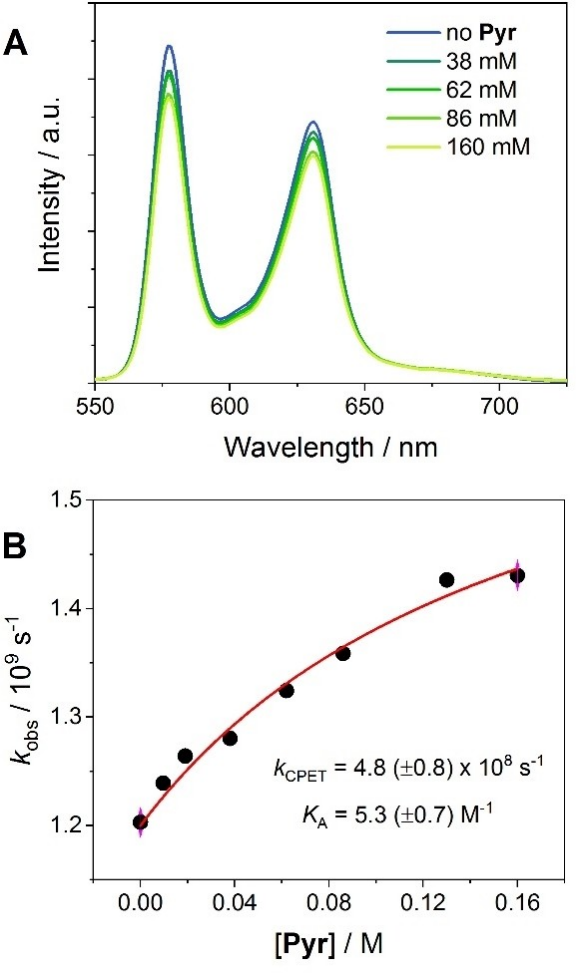
A) Relevant fluorescence spectra of **2** (excitation at 520 nm) in CH_2_Cl_2_ in the presence of 0–0.16 M pyrrolidine; B) plot of the singlet decay rate *k* (obtained by TC‐SPC) vs. pyrrolidine concentration (fitting of these experimental data according to [Eq. (1)]).

Differently from what observed in conjugate **1**, the fate of the singlet excited state of the SnOEP component in **2** is affected by the presence of pyrrolidine. Addition of the base causes indeed a weakening of the emission intensity accompanied by a corresponding decrease of the excited state lifetime (Figure 6). Interestingly, the quenching efficiency displays a saturation profile with respect to the amount of pyrrolidine added reaching a plateau (up to a maximum of ca. 15 %) at a base concentration of ≥∼0.1 M. Since pyrrolidine has negligible effects on the SnOEP singlet excited state decay (as observed with model compound **4**, Figure S35), this new quenching pathway can be univocally ascribed to the occurrence of a CPET process at the singlet level (Figure 4B). Application of the kinetic treatment used for CPET processes ([Eq. (1)])[^20^, ^30^] to the singlet excited state decay of **2** with pyrrolidine, using *k*_0_=1.2×10^9^ s^−1^ and neglecting the bimolecular process with rate constant *k_Q_*, results in a good fitting of the experimental data (Figure 6B). This treatment yields a rate constant of *k*_CPET_=4.8(±0.9)×10^8^ s^−1^ using the association constant of *K*_A_=5.3(±0.7) M^−1^.^[40]^

**Figure 7:**
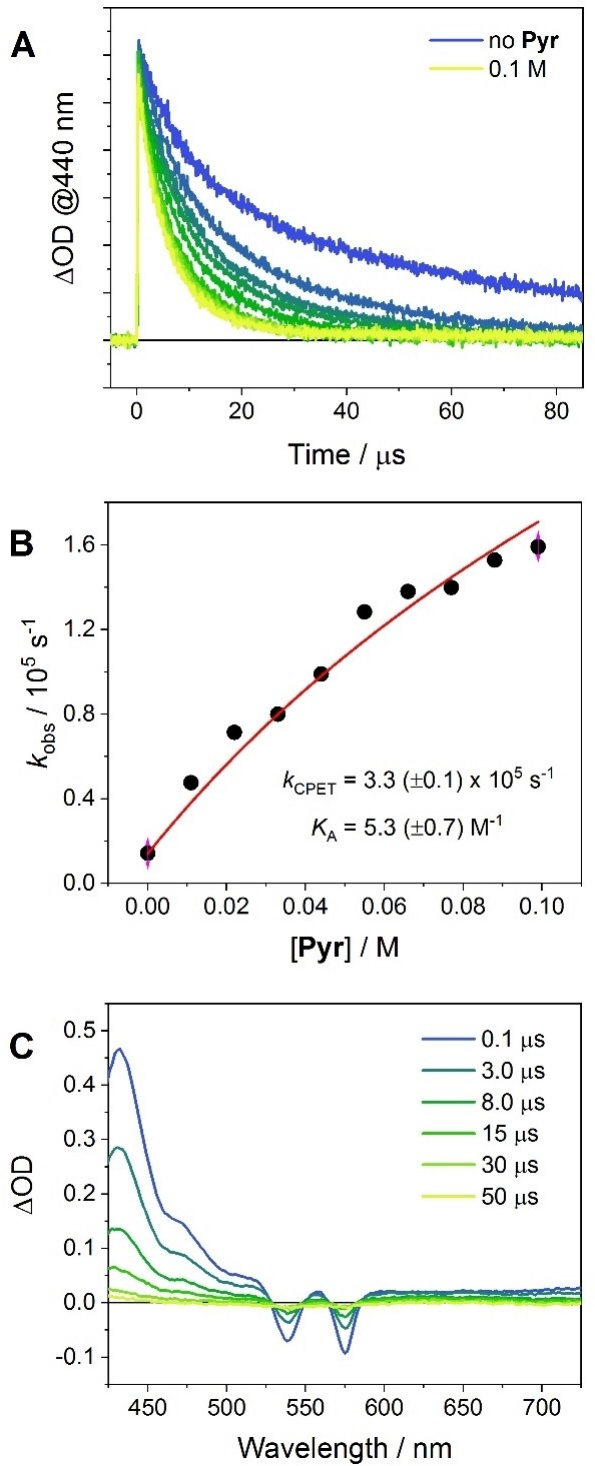
A) Relevant kinetic traces at 440 nm obtained by laser flash photolysis (excitation at 532 nm, 2 mJ) of **2** in N_2_‐purged CH_2_Cl_2_ in the presence of 0–0.1 M pyrrolidine; B) plot of the triplet decay rate *k* (obtained from a single exponential fitting of the decays in Fig. 7 A) vs. pyrrolidine concentration and fitting of the experimental data according to [Eq. (1)]; C) transient absorption spectra obtained by laser flash photolysis (excitation at 532 nm, 2 mJ) of **2** in N_2_‐purged CH_2_Cl_2_ in the presence of 0.1 M pyrrolidine.

The decay of the triplet excited state of the SnOEP component, populated by intersystem crossing from the singlet, is also affected by increasing addition of pyrrolidine, as measured near the triplet absorption maximum at 440 nm (Figure 7A).^[24]^ This behavior is characteristic of array **2** since addition of pyrrolidine to model compound **4** in CH_2_Cl_2_ has less pronounced effects on the triplet decay.^[20b]^ Also, as observed for the singlet excited state, the triplet decay in **2** with pyrrolidine can be well described using the kinetic formalism of [Eq. (1)] and points towards the occurrence of a CPET quenching mechanism (Figure 4B). Fitting of the data, using a *k*_0_=1.4×10^4^ s^−1^, a bimolecular rate constant of *k*_Q_=3.4×10^5^ M^−1^s^−1^,^[20b]^ and the *K*_A_=5.3(±0.7) M^−1^ estimated before (Figure 7B), results in a rate constant of *k*_CPET_=3.3(±0.1)×10^5^ s^−1^. Furthermore, the triplet excited state decay at 440 nm upon addition of pyrrolidine displays a kinetic isotope effect (KIE) of 1.2(±0.05) when **2** is investigated in CH_2_Cl_2_ in the presence of small aliquots of CH_3_OH or CD_3_OD (Figure S36).^[39]^ This value is comparable to the one found in the parent system **6**.^[20b]^ Thus, the observation of a KIE additionally corroborates the involvement of a concerted electron‐proton motion in the photoinduced quenching pathway.^[38]^ We would like to stress that small values of KIEs as well as the absence of any KIE are not in disagreement with such a PCET mechanism since the KIE is known to depend on many variables.[^6^, ^41^]

The spectral evolution of the transient absorption of **2** in CH_2_Cl_2_ in the presence of 0.13 M pyrrolidine has been then analyzed to gain further insight into the quenching process (Figure 7C). The transient spectrum immediately detected after 0.1 μs corresponds to the spectrum of the triplet excited state of the SnOEP component (absorption maximum at ca. 430 nm).^[24]^ This spectrum decays to the baseline without apparent formation of new transient signals (i. e., those corresponding to the radical pair state).[^28^, ^42^] This evidence thus suggests that, as observed in conjugate **1**, formation of the ^3^|SnOEP^.−^‐TrpN^.^| ⋅ ⋅ ⋅ ^+^HPyr diradical state is slower than the respective recombination. Within this hypothesis the products of the CPET process cannot accumulate to a measurable extent and be detected.

### Comparison with conjugates 5 and 6

The photophysical behavior of conjugates **1** and **2** in CH_2_Cl_2_ in the presence of pyrrolidine can be compared with that of the parent complexes **5** and **6** previously reported, featuring a *N‐*acetyl‐l‐tyrosine amino acidic residue in place of the tryptophan analog.^[20b]^ In particular, an interesting comparison between **1** and **5** emerges when the singlet excited state quenching is considered. As a matter of fact, although population of the radical pair state is always thermodynamically feasible from the singlet excited state of the SnTPP component, quenching occurs by ET‐only in the tryptophan case (**1**), whereas follows a CPET pathway in **5**.^[20]^ This is attributable to the lower oxidation potential of tryptophan compared to tyrosine that makes the charge transfer state of ET character accessible from the singlet excited state only in the tryptophan case. Accordingly, these results suggest that, even in the presence of a larger driving force, the enhanced mechanistic requirement (larger reorganization energy) in the coupled process seems to disfavor the photoinduced CPET pathway over the ET‐only. The larger rate constant measured for the singlet quenching in conjugate **1** (*k*_ET_=4.0×10^10^ s^−1^) compared to that estimated in **5** (*k*_CPET_=1.9×10^9^ s^−1^)^[20b]^ is consistent with this notion. On the other hand, when the population of a charge transfer state via ET is unfeasible on thermodynamic grounds, excited state quenching always follows a CPET mechanism in both **1** and **2** as observed in the parent compounds **5** and **6**. Indeed, quenching of the triplet excited state in **1** and of both singlet and triplet in **2** occurs via a CPET mechanism. Interestingly, the kinetics of the triplet CPET quenching process in **1** is comparable to the one measured in **5** (*k*_CPET_=4.0×10^6^ s^−1^ vs. *k*_CPET_=4.2×10^6^ s^−1^, respectively),^[20b]^ and the kinetics of triplet quenching in **2** to that of conjugate **6** (*k*_CPET_=3.3×10^5^ s^−1^ vs. *k*_CPET_=8.4×10^5^ s^−1^, respectively).^[20b]^ The observation of comparable kinetics for the photoinduced CPET pathways in both the tryptophan‐based (**1**, **2**) and the tyrosine‐based conjugates (**5** and **6**) strongly suggest that the SnP^.−^‐TrpN^.^ ⋅ ⋅ ⋅ ^+^HPyr and SnP^.−^‐TyrO^.^ ⋅ ⋅ ⋅ ^+^HPyr diradicals (with SnP being SnTPP or SnOEP) do possess similar energies in CH_2_Cl_2_ as the solvent. This can be attributed to the different redox and acid‐base behavior of the two amino acids: tyrosine is indeed more difficult to oxidize but easier to deprotonate than tryptophan^[3]^ so that the loss in ΔG_PT_ is compensated by a gain in ΔG_ET_ to yield a comparable ΔG_PCET_ for diradical formation upon excited state population. A subtle difference between conjugate **2** and **6** can be observed in the singlet quenching of the SnOEP unit, negligible in the latter, very weak but detectable in the former (*k*_CPET_=4.8×10^8^ s^−1^).^[20b]^ This can be possibly ascribed to the slightly different energetics of the forward CPET processes in either system as well as to different reorganization energies. Finally, akin to both complexes **5** and **6** and other related compounds,[^20b^, ^31^] also in conjugates **1** and **2** no accumulation of diradical species is attained as diradical recombination is always faster than formation. Nevertheless, it can be safely argued that radical pair recombination might occur through a stepwise ET‐PT process,[^20b^, ^31^] expected to be more favorable and kinetically easier than a concerted pathway.

## Conclusion

Two novel conjugates **1** and **2** have been synthesized and fully characterized including single crystal X‐ray analysis which evidences the H‐donor properties of the indole group of the tryptophan amino acidic residue. Their photophysical behavior has been examined in detail in CH_2_Cl_2_ in the presence of pyrrolidine as a base. Compound **1** is quenched at the singlet level via an ET‐only mechanism, whereas at the triplet level an efficient CPET quenching is observed. The faster ET quenching than CPET at the singlet level is consistent with a lower reorganization energy for the ET process with respect to the concerted pathway in spite of the relatively small driving force. In the case of **2** both excited states are quenched via a CPET mechanism. In all cases, no diradical species can be obtained for kinetic reasons as recombination is faster than the forward CPET step. In all cases the occurrence of CPET processes has been confirmed by a series of experimental results including kinetic modelling and determination of KIEs. Comparison with the parent conjugates featuring *N‐*acetyl‐l‐tyrosine as the redox active amino acid outlines the peculiar role of the tryptophan residue with respect to charge transfer reactions and its ability to undergo CPET processes provided that unfavorable competition with simple ET processes is avoided. These data add further mechanistic understanding on a simple yet complex process such as PCET and clearly evidence why Nature chose different amino acids to perform different charge transfer reactions in biochemical processes.

## Experimental Section

2,3,6,7,12,13,16,17‐Octaethylporphyrin (OEP) was purchased from Frontier Scientific. 5,10,15,20‐Tetraphenylporphyrin (TPP),^[43]^ *trans*‐dihydroxo(5,10,15,20‐tetraphenylporphyrinato)‐tin(IV) (SnTPP(OH)_2_), *trans*‐dihydroxo(2,3,6,7,12,13,16,17‐octaethylporphyrinato)‐tin(IV) (SnOEP(OH)_2_), *trans*‐dibenzoato(5,10,15,20‐tetraphenylporphyrinato)‐tin(IV) ((SnTPP(BA)_2_, **3**), and *trans*‐dibenzoato(2,3,6,7,12,13,16,17‐octaethylporphyrinato)‐tin(IV) ((SnOEP(BA)_2_, **4**) were prepared as reported earlier.[^20^, ^21^, ^44^, ^45^] Conjugates **1** and **2** were prepared as follows.

### *trans*‐Di(*N*‐acetyl‐l–tryptophanato)[5,10,15,20‐tetraphenyl‐porphyrinato]‐tin(IV) (1)^[20]^

*trans*‐Dihydroxo(5,10,15,20‐tetraphenylporphyrinato)‐tin(IV), SnTPP(OH)_2_, (17.8 mg, 0.023 mmol) was dissolved in 20 mL of CHCl_3_, and *N‐*acetyl‐l‐tryptophan (11.9 mg, 0.048 mmol) was then added to the clear violet solution. After stirring at reflux for 12 hours, the reaction mixture turned to a slightly colored solution with a purple precipitate. The solvent was removed on a rotary evaporator, the solid was dissolved in 10 mL of CHCl_3_ and *n*‐hexane was added to induce the precipitation of pure product as purple crystals (25.5 mg, 0.021 mmol, 91 % yield). ^1^H NMR (500 MHz, CDCl_3_) δ (ppm): 9.23 (s, 8H, Ha, ^4^ *J*(Sn–H)=15.0 Hz), 8.18 (d, *J*=7.1 Hz, 8H, Hb), 7.86 (t, *J*=7.6 Hz, 4H, Hd), 7.78 (t, *J*=7.5 Hz, 8H, Hc), 7.21 (s, 2H, Hl), 7.08 (d, *J*=8.2 Hz, 2H, Hm), 6.96 (t, *J*=7.6 Hz, 2H, Hn), 6.73 (t, *J*=7.5 Hz, 2H, Ho), 6.46 (d, *J*=7.9 Hz, 2H, Hp), 3.92 – 3.78 (m, 4H, Hf, Hi), 1.61 (dt, *J*=7.9, 4.6 Hz, 2H, He), 1.20 (dd, *J*=14.2, 5 Hz, 2H, Hh), 1.03 (s, 6H, Hg), 0.04 (dd, *J*=14.2, 4 Hz, 2H, Hh’). ^13^C NMR (125 MHz, CDCl_3_, from HSQC) δ (ppm): 133.43 (Cb), 131.66 (Ca), 127.19 (Cd), 125.97 (Cc), 119.99 (Cl), 118.87 (Ci), 117.90 (Cm), 116.59 (Cn), 108.94 (Ck), 49.42 (Ce), 22.90 (Ch), 21.31 (Cg).^119^Sn (186 MHz, CDCl_3_, from HMBC) δ (ppm): –630.8. Selected IR bands (cm^−1^, KBr pellets): 3430 (ῠ_NH_), 1650 (ῠ_C=O_). ESI‐MS (*m*/*z*) (negative mode) for C_70_H_52_N_8_O_6_Sn_1_ [**1** − 2H]: 1221.3, found 1221.2. Single crystals suitable for X‐ray diffraction were obtained by slow diffusion of *n*‐hexane over a concentrated solution of **1** in CHCl_3_.

### *trans*‐Di(*N‐*acetyl‐l–tryptophanato)[2,3,6,7,12,13,16,17‐octa‐ethylporphyrinato]‐tin(IV) (2)

*trans*‐Dihydroxo (2,3,6,7,12,13,16,17‐octaethylporphyrinato)‐tin(IV), SnOEP(OH)_2_, (21 mg, 0.031 mmol) was dissolved in 20 mL of CHCl_3_, and *N‐*acetyl‐l‐tryptophan (15.8 mg, 0.064 mmol) was then added to the clear violet solution. After stirring at reflux for 12 hours, the reaction mixture turned to a slightly colored solution with a purple precipitate. The solvent was removed on a rotary evaporator, the solid was washed with 10 mL of water, filtered and dried under vacuum (32.2 mg, 0.028 mmol, 92 % yield). ^1^H NMR (500 MHz, CDCl_3_) δ (ppm): 10.58 (s, 4H, Ha), 7.17 (br, 2H, Hi), 7.03 (d, *J*=8.1 Hz, 2H, Hj), 6.89 (t, *J*=7.4 Hz, 2H, Hk), 6.68 (t, *J*=7.4 Hz, 2H, Hl), 6.31 (d, *J*=7.9 Hz, 2H, Hm), 4.26 (q, *J*=7.7 Hz, 16H, Hb), 3.92 (d, *J*=2 Hz, 2H, Hh), 3.67 (d, *J*=7.8 Hz, 2H, He), 2.01 (t, *J*=7.7 Hz, 24H, Hc), 1.24 (dt, *J*=8.0, 4.7 Hz 2H, Hd), 0.96− 0.82 (m, 8H, Hf, Hg) −0.49 (dd, *J*=14.2, 4.4 Hz, 2H, Hg’). ^13^C NMR (125 MHz, CDCl_3_, from HSQC) δ (ppm): 121.29 (Ck), 120.28 (Ch), 118.75 (Cl), 118.05 (Cm), 110.28 (Cj), 97.47 (Ca), 50.87 (Cd), 24.44 (Cg), 22.51 (Cf), 20.09 (Cb), 18.57 (Cc). ^119^Sn (186 MHz, CDCl_3_, from HMBC) δ (ppm): –633.9. Selected IR bands (cm^−1^, KBr pellets): 3414 (ῠ_NH_), 1650 (ῠ_C=O_). ESI‐MS (*m*/*z*) (negative mode) for C_62_H_68_N_8_O_6_Sn_1_ [**2** − 2H]: 1141.4, found 1141.4.

The products have been thoroughly characterized using NMR, ESI‐MS, and IR analyses (see Supporting Information for details). Deposition Numbers 2051475 (for **1**) and 2051772 (for **4**) contain the supplementary crystallographic data for this paper. These data are provided free of charge by the joint Cambridge Crystallographic Data Centre and Fachinformationszentrum Karlsruhe Access Structures service www.ccdc.cam.ac.uk/structures.

## Conflict of interest

The authors declare no conflict of interest.

## Supporting information

As a service to our authors and readers, this journal provides supporting information supplied by the authors. Such materials are peer reviewed and may be re‐organized for online delivery, but are not copy‐edited or typeset. Technical support issues arising from supporting information (other than missing files) should be addressed to the authors.

Supplementary
