# Supplementary material for: Photoinduced Electron vs. Concerted Proton Electron Transfer Pathways in SnIV (l‐Tryptophanato)2 Porphyrin Conjugates
Source: Chemistry. 2021 May 2;27(29):7872–81. doi: 10.1002/chem.202005487 (PMC8252543; doi:10.1002/chem.202005487)
Supplement: Supplementary file 1 — Supplementary [file CHEM-27-7872-s001.pdf]

Chemistry–A European Journal

Supporting Information

Photoinduced Electron vs. Concerted Proton Electron Transfer Pathways in Sn^{IV} (L-Tryptophanato)$_2$ Porphyrin Conjugates

Mirco Natali,* Agnese Amati, Nicola Demitri, and Elisabetta Iengo*

Table of contents

**S1.**	Experimental section	p. S2
**S2.**	Synthesis and characterization	p. S6
**S3.**	Crystallographic data	p. S11
**S4.**	Electrochemical characterization	p. S19
**S5.**	Photophysical characterization	p. S22
**S6.**	Energy level diagrams	p. S37
**S7.**	References of the Supporting Information	p. S39

S1. Experimental section

Materials and methods. Chemicals were purchased from Sigma-Aldrich or Alfa Aesar and used without further purification, unless otherwise stated. Solvents for spectroscopic measurements were of spectroscopic grade, all other chemicals were of reagent grade quality, and used as received. Deuterated chloroform was purchased from Sigma-Aldrich.

NMR. Mono- and bi-dimensional NMR experiments (^{1}H, H-H COSY, H-C COSY, H-Sn HMBC) were recorded on a Varian 500 spectrometer (operating at 500 MHz for ^{1}H, 125 MHz for ^{13}C, and 186 MHz for ^{119}Sn). All spectra were run at room temperature; ^{1}H and ^{13}C chemical shifts were referenced to the peak of residual non-deuterated solvents: δ (ppm) = 7.26 and δ (ppm) = 77.16, respectively, for $CDCl_3$. ^{119}Sn chemical shifts were referenced to the internal standards tetramethyltin at 0.00 ppm.

ESI-MS. Electrospray ionization mass spectrometry (ESI-MS) measurements were performed on a Perkin–Elmer APII spectrometer at 5600 eV.

IR. Infrared spectra were recorded on a Perkin-Elmer FT-IR 2000 spectrometer in the transmission mode and the samples were prepared as KBr pellets.

Electrochemistry. Cyclic Voltammetry (CV) measurements were carried out with a PC-interfaced *Eco Chemie Autolab/Pgstat 30* Potentiostat. Nitrogen-purged 10^{-3} M sample solutions in dichloromethane, containing 0.1 M $TBAPF_6$ (Tetrabutylammonium hexafluorophosphate, Fluka, electrochemical grade, 99%, dried in an oven), were used. A conventional three-electrode cell assembly was adopted: a saturated calomel electrode (SCE Amel) and a platinum electrode, both separated from test solution by a glass frit, were used as reference and counter (CE) electrodes,

respectively; a glassy carbon (GC) electrode was used as the working electrode (WE). Ferrocene was added and used as an internal standard to which the potentials were referred.

UV-Vis spectrophotometric analysis. UV-Vis absorption spectra were recorded on a *Cary 300 UV-Vis* (*Agilent Technologies*) spectrophotometer.

Spectrofluorimetric analysis. Emission spectra were taken on an *Edinburgh Instrument* spectrofluorometer equipped with a 900 W Xe arc lamp as excitation source, a photomultiplier tube, and an InGaAs detector for the visible and the NIR detection, respectively.

Time-correlated single photon counting. Fluorescence lifetimes were measured using a TC-SPC apparatus (*PicoQuant Picoharp 300*) equipped with subnanosecond LED sources (600 nm for **1,3** and 380 nm for **2,4**, 500-700 ps pulse width) powered by a *PicoQuant PDL 800-B* variable (2.5-40 MHz) pulsed power supply. The decays were analyzed by means of *PicoQuant FluoFit* Global Fluorescence Decay Analysis Software.

Nanosecond transient absorption spectroscopy. Nanosecond transient measurements were performed with a custom laser spectrometer comprised of a *Continuum Surelite II* Nd:YAG laser (FWHM 6-8 ns) with frequency doubled (532 nm, 330 mJ) option, an *Applied Photophysics* xenon light source including a mod. 720 150W lamp housing, a mod. 620 power-controlled lamp supply and a mod. 03 –102 arc lamp pulser. Laser excitation was provided at 90° with respect to the white light probe beam. Light transmitted by the sample was focused onto the entrance slit of a 300 mm focal length *Acton SpectraPro 2300i* triple grating, flat field, double exit monochromator equipped with a photomultiplier detector (*Hamamatsu R3896*) and a *Princeton Instruments PIMAX II* gated intensified CCD camera, using an *RB Gen II* intensifier, a ST133 controller and a PTG pulser. Signals from the photomultiplier (kinetic traces) were processed by means of a *TeledyneLeCroy*

604Zi (400 MHz, 20 GS/s) digital oscilloscope. Transient measurements were performed in dichloromethane solutions after purging with N_2 for ca 10 minutes before each experiment.

Ultrafast spectroscopy. Ultrafast spectroscopy experiments in the ps time-range were performed using a pump-probe setup based on a Spectra-Physics Hurricane Ti:sapphire laser source (FWHM ca. 130 fs) and an Ultrafast Systems Helios spectrometer. Excitation pulses were generated *via* an SHG option (400 nm). Probe pulses were obtained by continuum generation on a sapphire plate (useful spectral range: 450-750 nm). Effective time resolution *ca. 2*00 fs, temporal window of the optical delay stage 0-1000 ps. The time-resolved spectral data were deconvoluted to correct for spectral chirp and thus analysed with the Ultrafast Systems Surface Explorer Pro software.

Crystal structure determination. Data collections were performed at the X-ray diffraction beamline (XRD1) of the Elettra Synchrotron, Trieste (Italy).[S1] CCDC 2051475 and 2051772 contain the crystallographic data for conjugate **1** and model compound **4**, respectively. These data can be obtained free of charge from The Cambridge Crystallographic Data Centre via https://www.ccdc.cam.ac.uk/structures. The obtained crystals were dipped in NHV oil (Jena Bioscience, Jena, Germany) and mounted on the goniometer head with kapton loops (MiTeGen, Ithaca, USA). Complete datasets were collected at 100 K (nitrogen stream supplied through an Oxford Cryostream 700 - Oxford Cryosystems Ltd., Oxford, United Kingdom) through the rotating crystal method. Data were acquired using a monochromatic wavelength of 0.700 Å on a Pilatus 2M hybrid-pixel area detector (DECTRIS Ltd., Baden-Daettwil, Switzerland). The diffraction data were indexed and integrated using XDS.[S2] The structures were solved by the dual space algorithm implemented in SHELXT.[S3] Fourier analysis and refinement were performed by the full-matrix least-squares methods based on F^2 implemented in SHELXL (Version 2017/1).[S4] The Coot program was used for modelling.[S4] Anisotropic thermal motion refinement have been used for all atoms. Hydrogen atoms were included at calculated positions with isotropic $U_{factors} = 1.2 \cdot U_{eq}$ or

$U_{factors}$ = 1.5·U_{eq} for methyl and hydroxyl groups (U_{eq} being the equivalent isotropic thermal factor of the bonded non hydrogen atom). One full **1** complex has been found in the crystallographic asymmetric unit (Figure 1). Two chloroform molecules have been modelled in crystal cavities, defined by the porphyrin area not occupied by the bound tryptophan residues. Geometric parameter restrains (DFIX and DANG) have been applied on solvent molecules. Refined Flack parameters confirms the expected amino acid chirality (L-aa). Half compound **4** was found in the crystallographic asymmetric unit (Figure S14). Pictures were prepared using Ortep3 and Pymol software.[S5,S6] Essential crystal and refinement data are reported in the Supporting Information.

S2. Synthesis and characterization

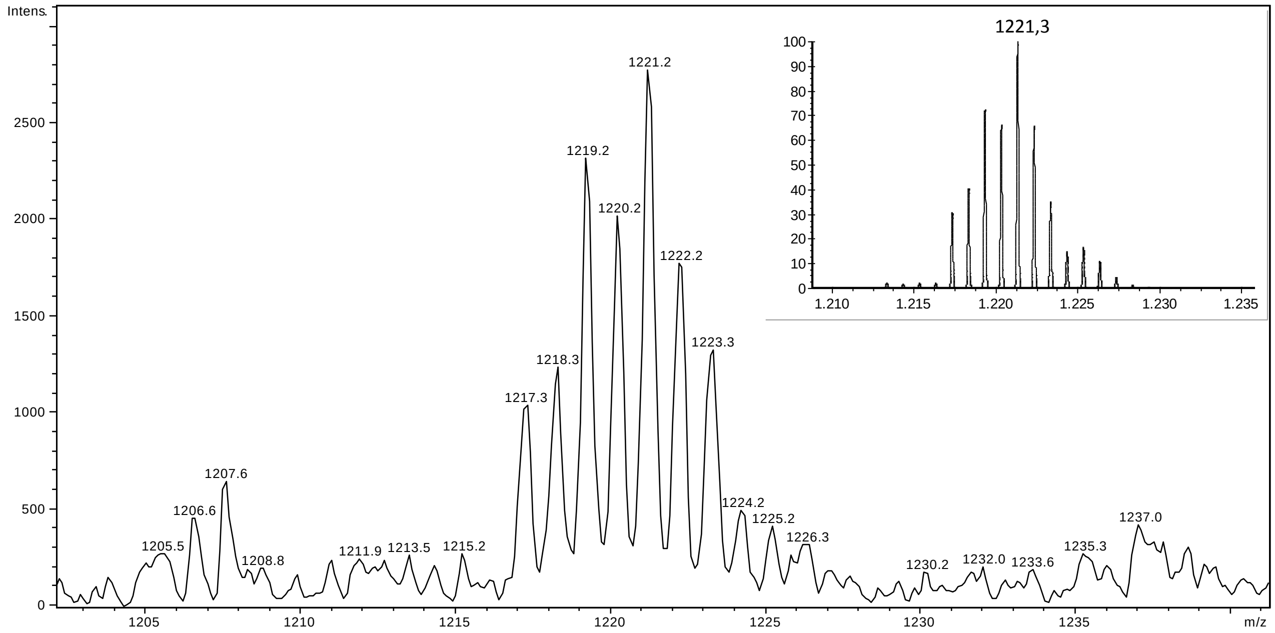

Figure S1. ESI-MS (*m/z*) (negative mode) of **1**: experimental and calculated (program IsoPro3, inset) isotopic distributions of the [**1** − 2H]$^{\bullet-}$ peak centered at *m/z* 1221.2.

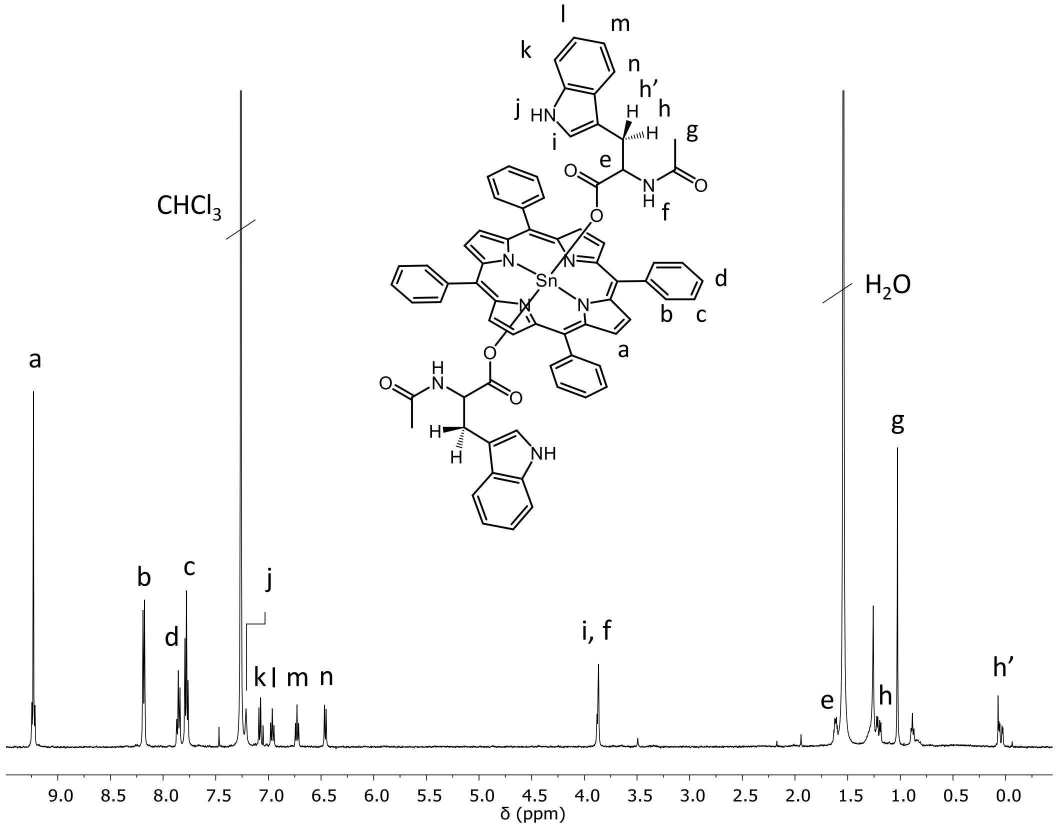

Figure S2. ^{1}H NMR spectrum ($CDCl_3$) of **1**.

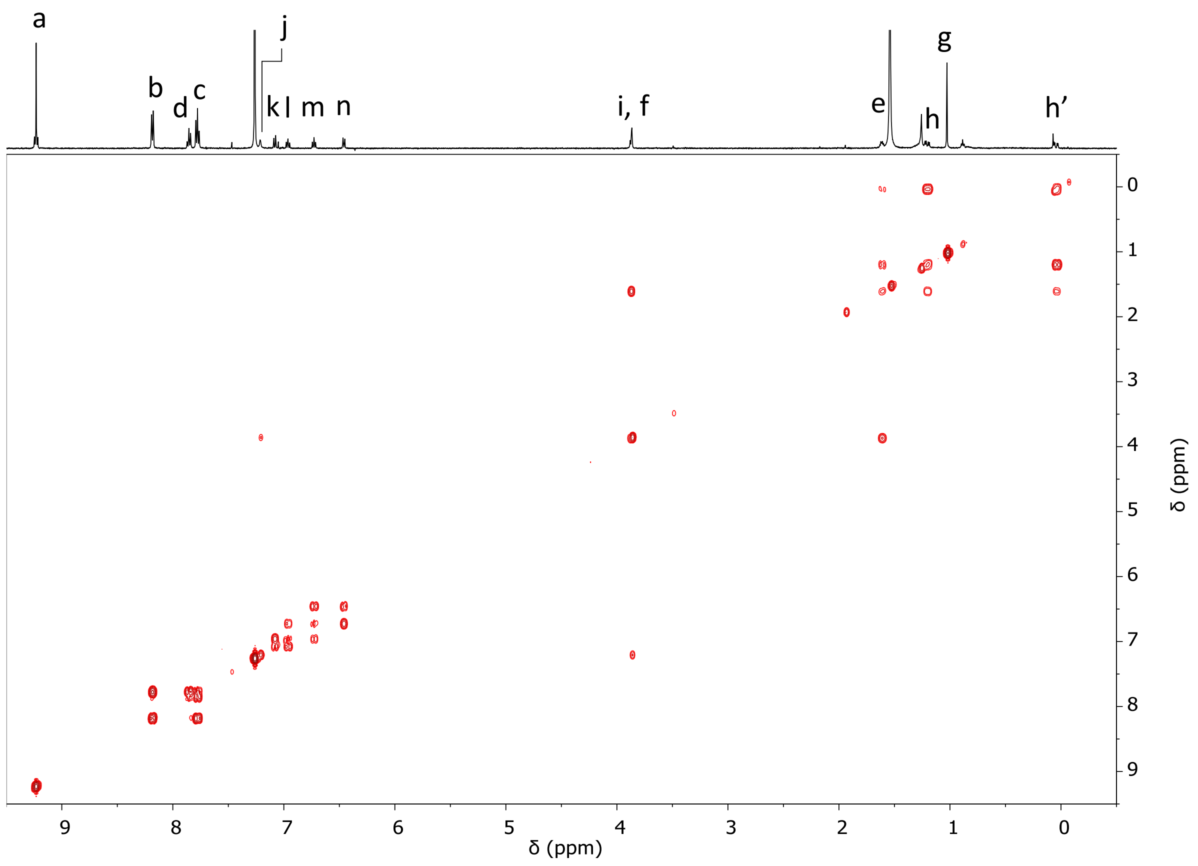

Figure S3. H–H COSY spectrum ($CDCl_3$) of **1**.

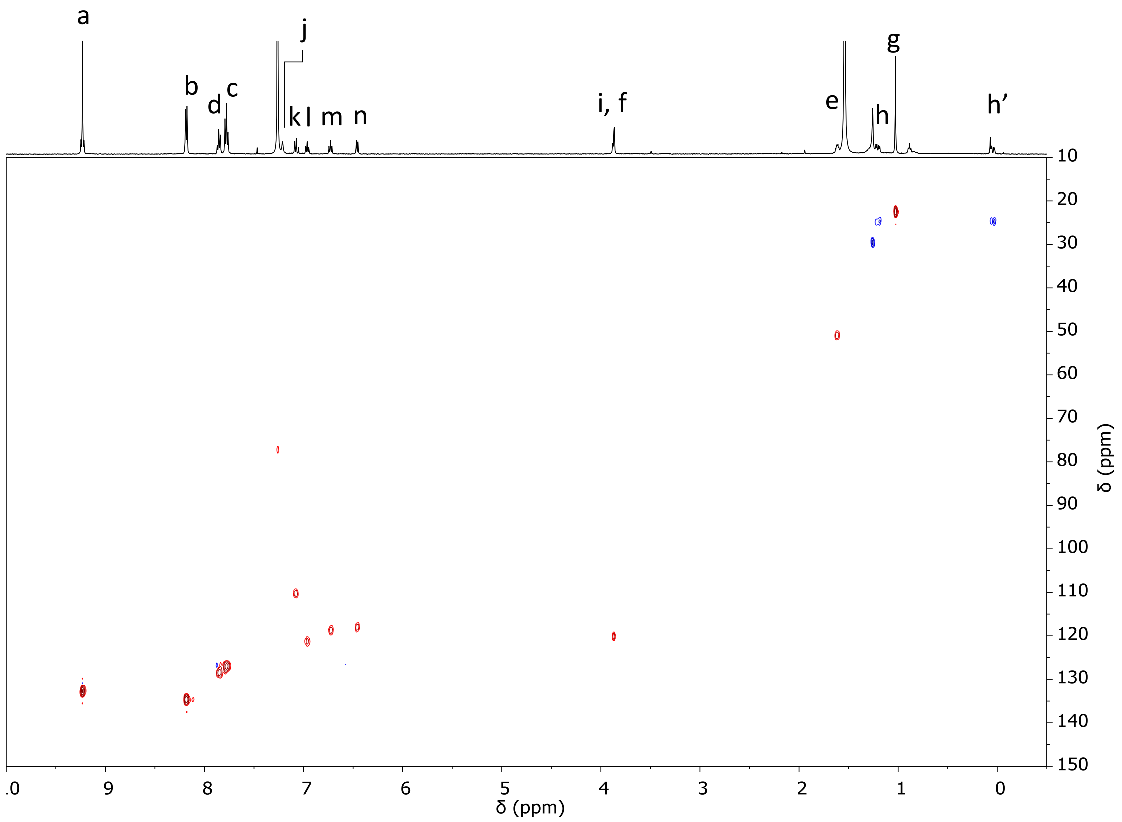

Figure S4. H–C COSY spectrum ($CDCl_3$) of **1**.

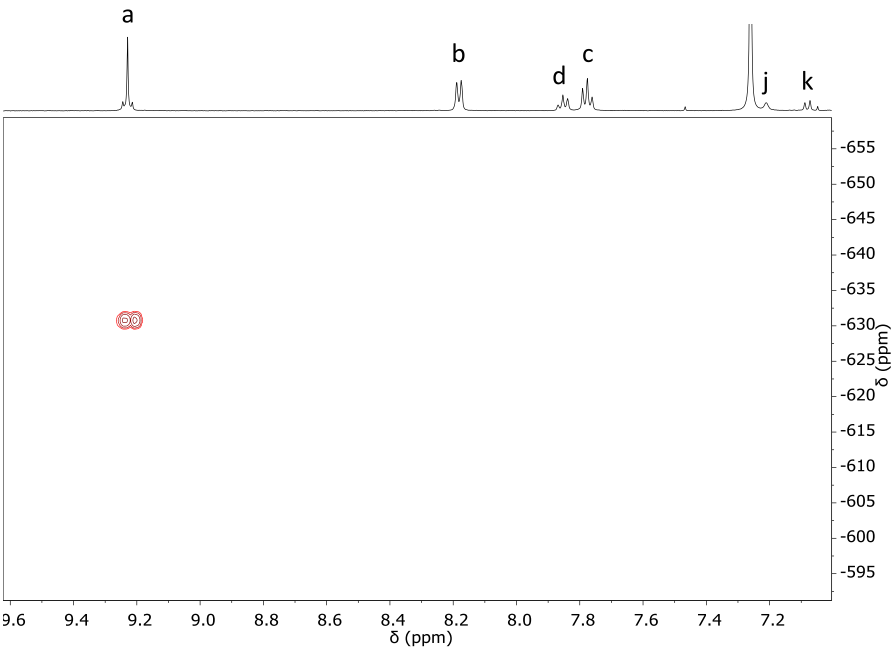

Figure S5. H–Sn HMBC spectrum ($CDCl_3$) of **1**.

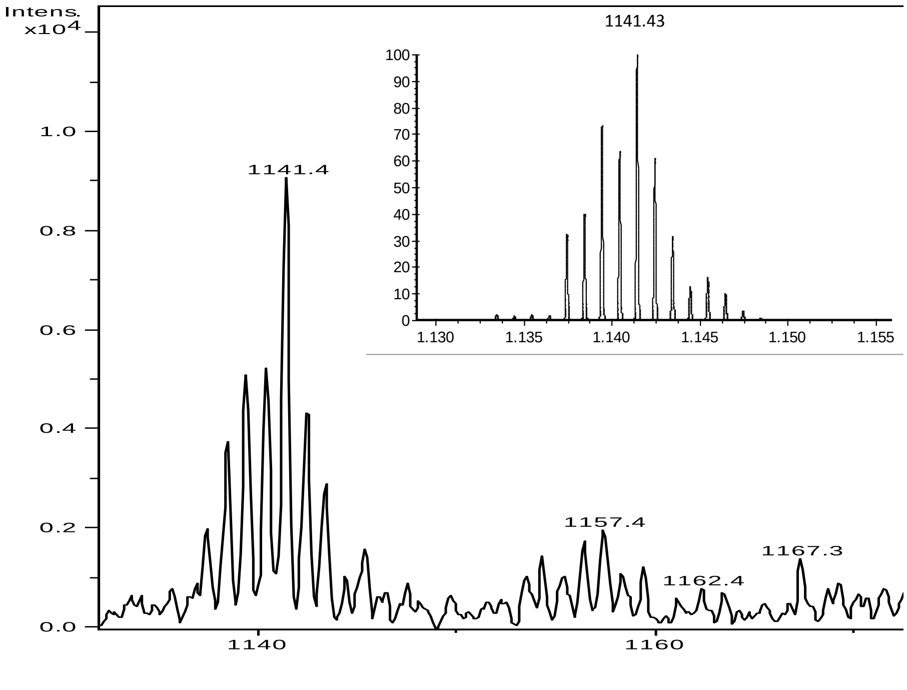

Figure S6. ESI-MS (*m/z*) (negative mode) of **2**: experimental and calculated (program IsoPro3, inset) isotopic distributions of the [**2** − 2H]$^{\cdot-}$ peak centered at *m/z* 1141.4.

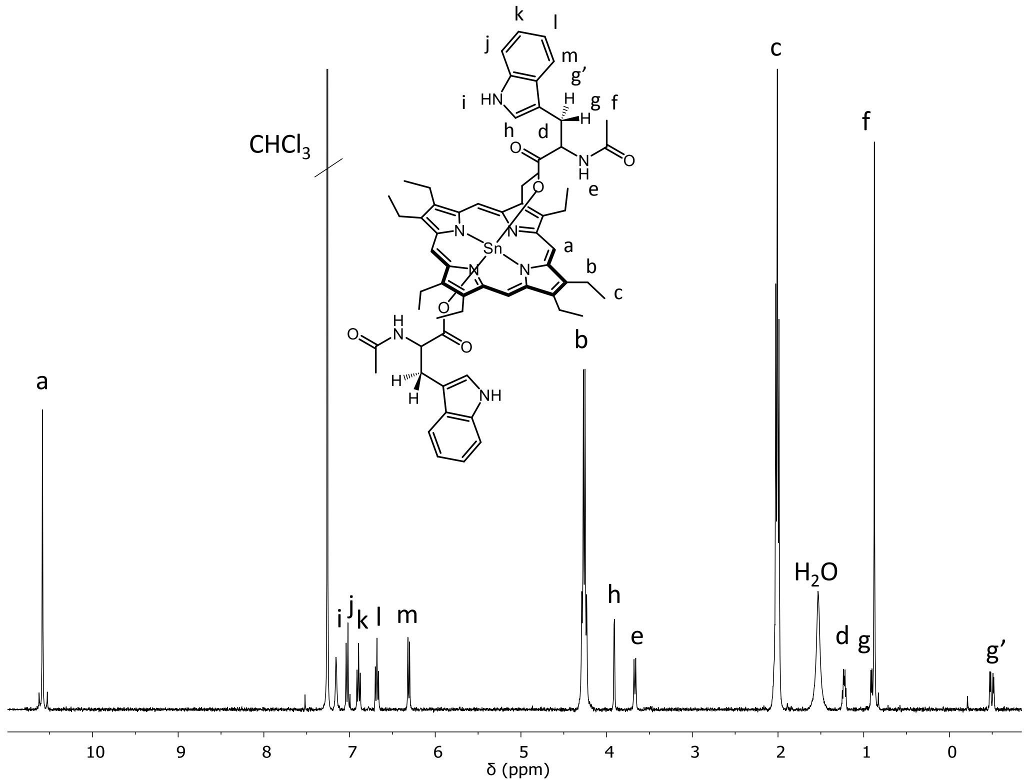

Figure S7. ^{1}H NMR spectrum ($CDCl_3$) of **2**.

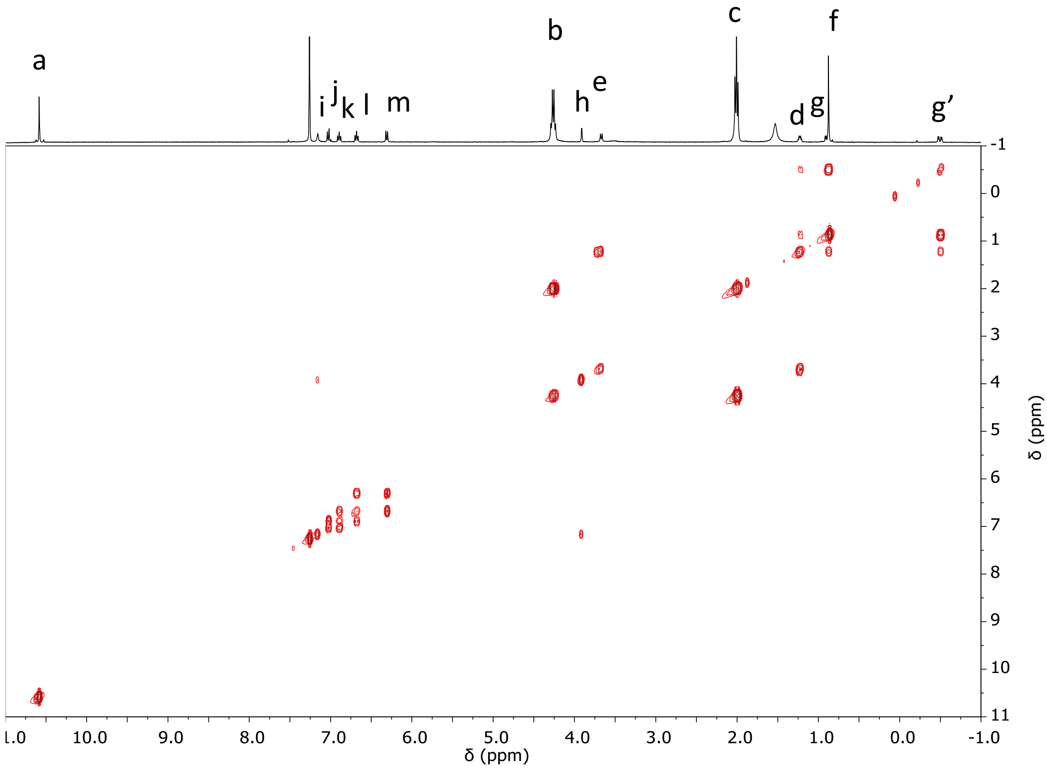

Figure S8. H–H COSY spectrum ($CDCl_3$) of **2**.

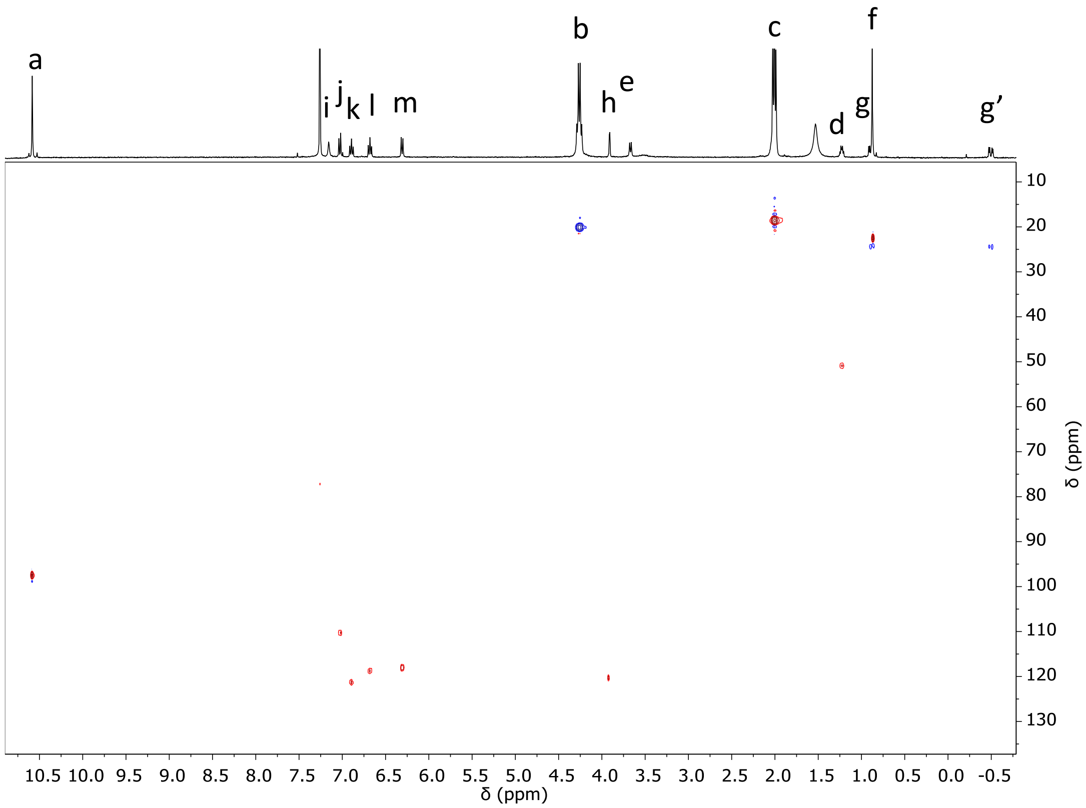

Figure S9. H–C COSY spectrum ($CDCl_3$) of **2**.

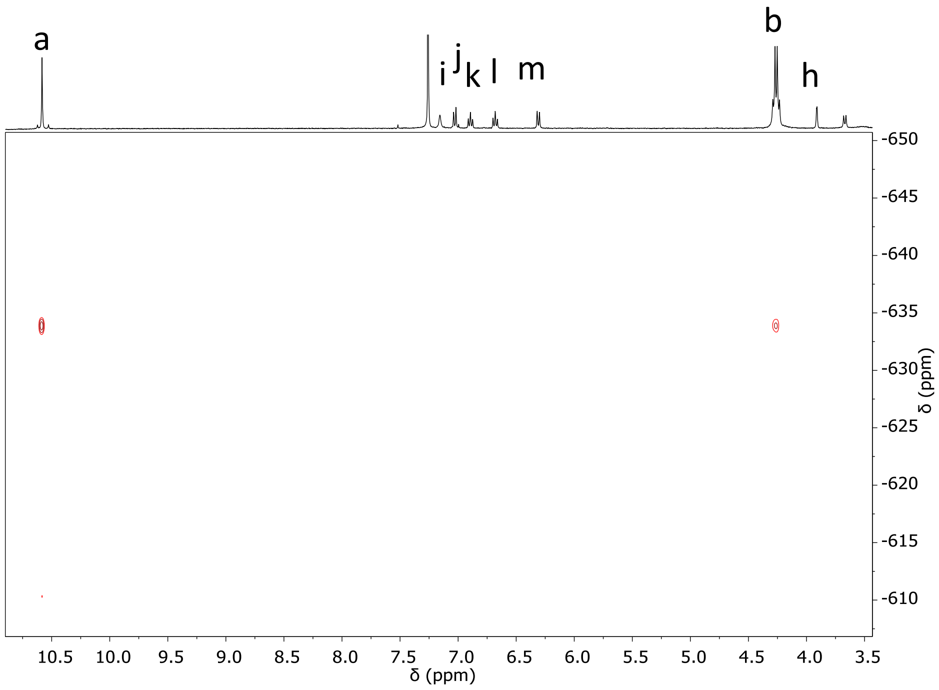

Figure S10. H–Sn HMBC spectrum ($CDCl_3$) of **2**.

S3. Crystallographic data

Table S1. Crystallographic data and refinement details for **1** and **4**.

	**1**·$2CHCl_3$ [$C_{70}H_{54}N_8O_6Sn\cdot 2CHCl_3$]	**4** [$C_{50}H_{54}N_4O_4Sn$]
CCDC Number	2051475	2051772
Chemical Formula	$C_{72}H_{56}Cl_6N_8O_6Sn$	$C_{50}H_{54}N_4O_4Sn$
Formula weight (g/mol)	1460.63	893.66
Temperature (K)	100(2)	100(2)
Wavelength (Å)	0.700	0.700
Crystal system	Monoclinic	Monoclinic
Space Group	$P2_1$	$I\ 2/a$
Unit cell dimensions	a = 11.326(2)Å	a = 10.573(2) Å
	b = 13.893(3)Å	b = 21.244(4) Å
	c = 21.054(4)Å	c = 18.441(4) Å
	β = 99.67(3)°	β = 92.19(3)°
Volume (Å^3)	3265.8(12)	4139.1(14)
Z	2	4
Density (calculated) (g·cm^{-3})	1.485	1.434
Absorption coefficient (mm^{-1})	0.661	0.635
F(000)	1488	1856
Crystal size (mm^3)	0.03 x 0.03 x 0.01	0.10 x 0.08 x 0.03
Crystal habit	Pale yellow thick rods	Pale yellow thick rods
Theta range for data collection	0.97° to 30.98°	1.44° to 30.96°
Resolution (Å)	0.68	0.68
Index ranges	$-16 \le h \le 16$	$-15 \le h \le 15$
	$-20 \le k \le 20$	$-31 \le k \le 31$
	$-30 \le l \le 30$	$-25 \le l \le 25$
Reflections collected	36815	40455
Independent reflections (data with $I>2\sigma(I)$)	20000 (19253)	6681 (6429)
Data multiplicity (max resltn)	3.27 (2.83)	5.82 (5.01)
$I/\sigma(I)$ (max resltn)	37.89 (23.91)	21.67 (18.82)
R_{merge} (max resltn)	0.0328 (0.0519)	0.0688 (0.0700)
Data completeness (max resltn)	97.1% (91.7%)	97.2% (92.3%)
Refinement method	Full-matrix least-squares on F^2	Full-matrix least-squares on F^2
Data / restraints / parameters	20000/ 13 / 877	6681 / 0 / 273
Goodness-of-fit on F^2	1.053	1.033
Δ/σ_{max}	0.009	0.001
Final R indices [$I>2\sigma(I)$]^a	R_1 = 0.0374, wR_2= 0.0996	R_1 = 0.0369, wR_2 = 0.1024
R indices (all data)^a	R_1 = 0.0389, wR_2= 0.1008	R_1 = 0.0376, wR_2 = 0.1032
Flack x parameter	-0.002(6)	N.A.
Largest diff. peak and hole(e·Å^{-3})	1.503 and -1.373	1.166 and -0.688
R.M.S. deviation from mean(e·Å^{-3})	0.087	0.126

$^aR_1 = \Sigma\,||Fo|-|Fc||\ /\ \Sigma|Fo|$, $wR_2 = \{\Sigma\,[w(Fo^2 - Fc^2)^2]/\ \Sigma\,[w(Fo^2)^2]\}^{½}$

Table S2. Selected bond distances and angles (Å and °) for tin coordination sphere in **1** and **4**. The coordination spheres of **3**[S7] and **5**[S8] are reported for comparison.

**1**				**4**			
**Distances**	**(Å)**	**Angles**	**(°)**	**Distances**	**(Å)**	**Angles**	**(°)**
Sn_1-NBD_2	2.088(3)	NBD_2-Sn_1-NAW_2	90.99(10)	Sn-N1	2.091(1)	N1-Sn-N2	90.15(4)
Sn_1-NAW_2	2.089(3)	NBD_2-Sn_1-OXT_3	85.40(10)	Sn-N2	2.087(1)	N1-Sn-O2	83.72(6)
Sn_1-NAP_2	2.088(2)	NBD_2-Sn_1-NAP_2	178.85(11)	Sn-N1_#1	2.091(1)	N1-Sn-N1_#1	180
Sn_1-NAF_2	2.099(2)	NBD_2-Sn_1-OXT_5	92.91(10)	Sn-N2_#1	2.087(1)	N1-Sn-O2_#1	96.28(6)
Sn_1-OXT_3	2.104(2)	NBD_2-Sn_1-NAF_2	89.21(10)	Sn-O2	2.057(1)	N1-Sn-N2_#1	89.85(4)
Sn_1-OXT_5	2.095(2)	NAW_2-Sn_1-OXT_3	92.15(10)	Sn-O2_#1	2.057(1)	N2-Sn-O2	86.95(5)
		NAW_2-Sn_1-NAP_2	89.64(10)			N2-Sn-N1_#1	89.85(4)
		NAW_2-Sn_1-OXT_5	92.56(10)			N2-Sn-O2_#1	93.05(5)
		NAW_2-Sn_1-NAF_2	179.56(11)			N2-Sn-N2_#1	180
		OXT_3-Sn_1-NAP_2	93.62(10)			O2-Sn-N1_#1	96.28(6)
		OXT_3-Sn_1-OXT_5	175.02(9)			O2-Sn-O2_#1	180
		OXT_3-Sn_1-NAF_2	87.47(10)			O2-Sn-N2_#1	93.05(5)
		NAP_2-Sn_1-OXT_5	88.02(10)			N1_#1-Sn-O2_#1	83.72(6)
		NAP_2-Sn_1-NAF_2	90.15(10)			N1_#1-Sn-N2_#1	90.15(4)
		OXT_5-Sn_1-NAF_2	87.82(10)			O2_#1-Sn-N2_#1	86.95(5)

Symmetry transformations used to generate equivalent atoms: #1: -x+1/2, -y+1/2, -z+1/2

**3** [S1a]				**5** [S2]			
**Distances**	**(Å)**	**Angles**	**(°)**	**Distances**	**(Å)**	**Angles**	**(°)**
Sn1-N1	2.088(5)	N1-Sn1-N2	90.21(24)	Sn_1-NBD_2	2.075(5)	NBD_2-Sn_1-NAW_2	90.01(18)
Sn1-N2	2.085(8)	N1-Sn1-O2	86.21(24)	Sn_1-NAW_2	2.082(4)	NBD_2-Sn_1-OXT_3	97.21(16)
Sn1-N1_#1	2.088(5)	N1-Sn1-N1_#1	180	Sn_1-NAP_2	2.094(4)	NBD_2-Sn_1-NAP_2	178.88(25)
Sn1-N2_#1	2.085(8)	N1-Sn1-O2_#1	93.79(24)	Sn_1-NAF_2	2.106(5)	NBD_2-Sn_1-OXT_5	93.19(16)
Sn1-O2	2.055(5)	N1-Sn1-N2_#1	89.79(24)	Sn_1-OXT_3	2.089(3)	NBD_2-Sn_1-NAF_2	89.61(18)
Sn1-O2_#1	2.055(5)	N2-Sn1-O2	88.26(24)	Sn_1-OXT_5	2.102(3)	NAW_2-Sn_1-OXT_3	89.99(16)
		N2-Sn1-N1_#1	89.79(24)			NAW_2-Sn_1-NAP_2	90.27(17)
		N2-Sn1-O2_#1	91.74(24)			NAW_2-Sn_1-OXT_5	93.18(15)
		N2-Sn1-N2_#1	180			NAW_2-Sn_1-NAF_2	179.36(25)
		O2-Sn1-N1_#1	93.79(24)			OXT_3-Sn_1-NAP_2	83.87(16)
		O2-Sn1-O2_#1	180			OXT_3-Sn_1-OXT_5	169.13(10)
		O2-Sn1-N2_#1	91.74(24)			OXT_3-Sn_1-NAF_2	90.56(16)
		N1_#1-Sn1-O2_#1	86.21(24)			NAP_2-Sn_1-OXT_5	85.72(15)
		N1_#1-Sn1-N2_#1	90.21(24)			NAP_2-Sn_1-NAF_2	90.10(17)
		O2_#1-Sn1-N2_#1	88.26(24)			OXT_5-Sn_1-NAF_2	86.33(16)

Symmetry transformations used to generate equivalent atoms: #1: -x, -y, -z

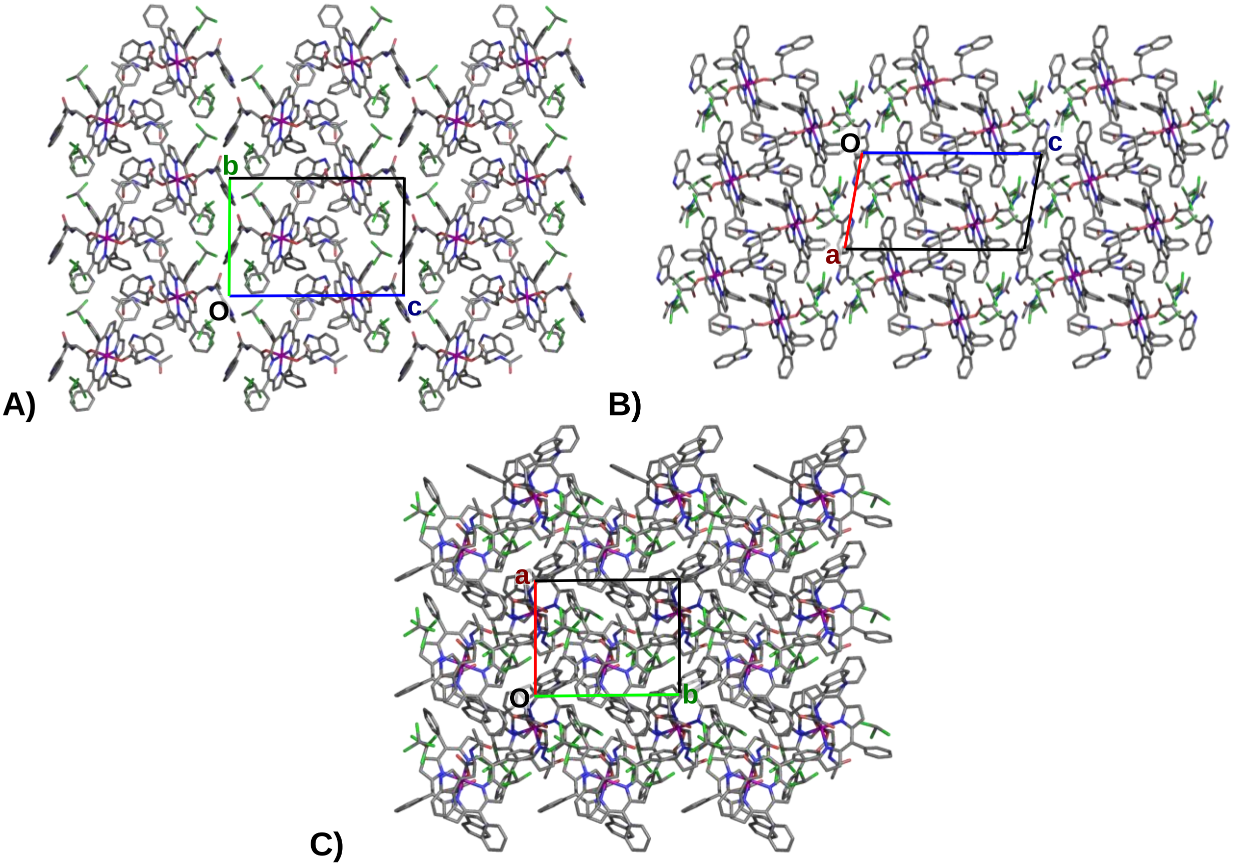

Figure S11. Views of **1** along crystallographic A) *a*, B) *b* and C) *c* axis. Hydrogens omitted for clarity.

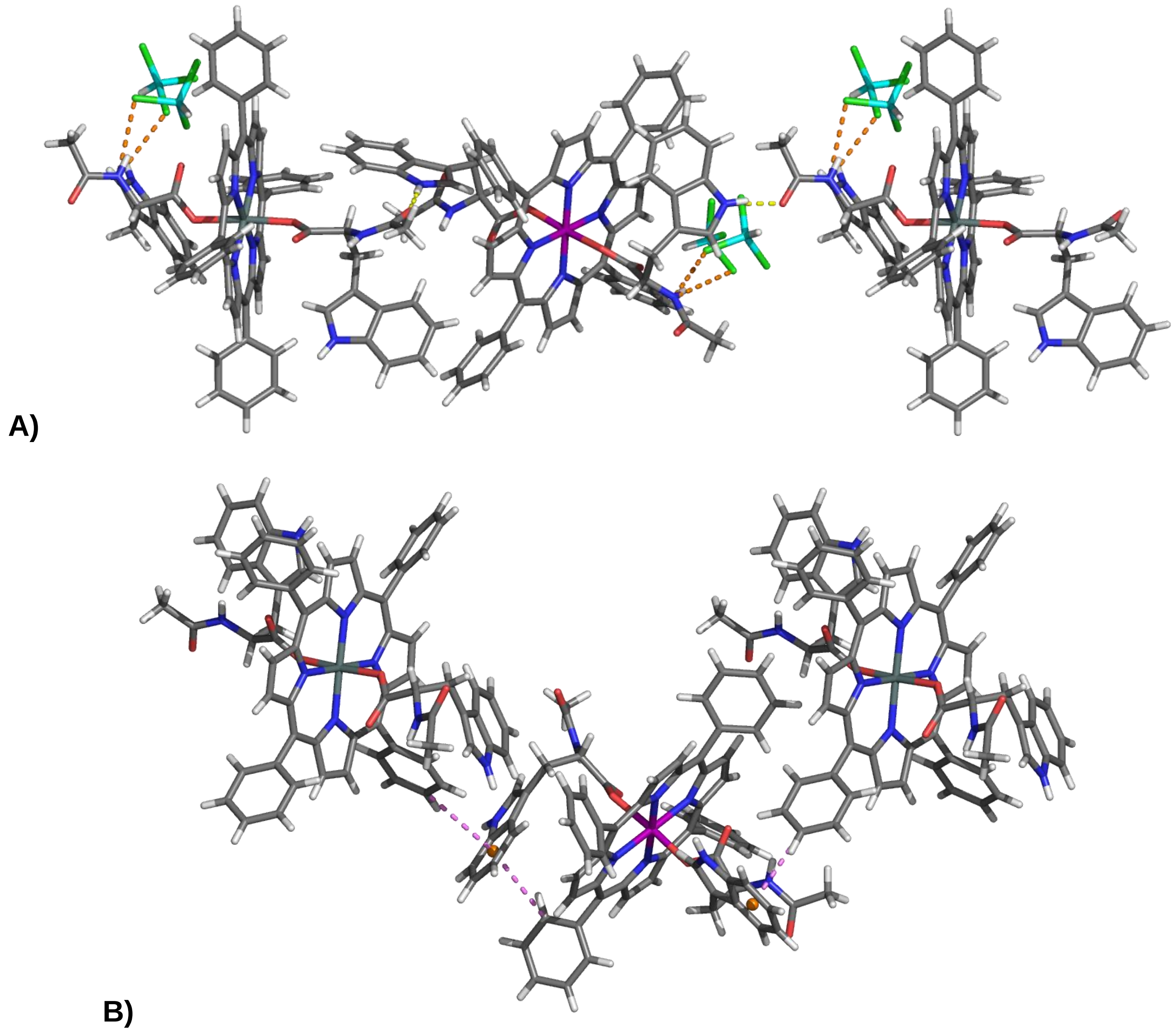

Figure S12. Crystal packing excerpts of **1**. A) The tin-porphyrin conjugates are connected through hydrogen bonds between the amino acid carbonyl acetylated terminals and–NH indole side chains, of adjacent adducts, giving rise to linear arrays ($d_{NH\cdots OC}$ = 2.829(5) Å and $d_{NH\cdots OC}$ = 2.862(5) Å, in yellow dashed sticks). Crystallization chloroform molecules (light blu sticks), are connected through hydrogen bonds by the terminal acetylated nitrogens ($d_{NH\cdots Cl}$ = 3.68(1) Å, represented with orange dashed sticks). B) CH•••π intra- and intermolecular interactions can be found, involving peripheral porphyrin phenyl hydrogens and indole rings (intramolecular $d_{CH\cdots\pi}$ = 3.510(4)Å - intermolecular $d_{CH\cdots\pi}$ = 3.641(5)Å, shown with violet dashed sticks).

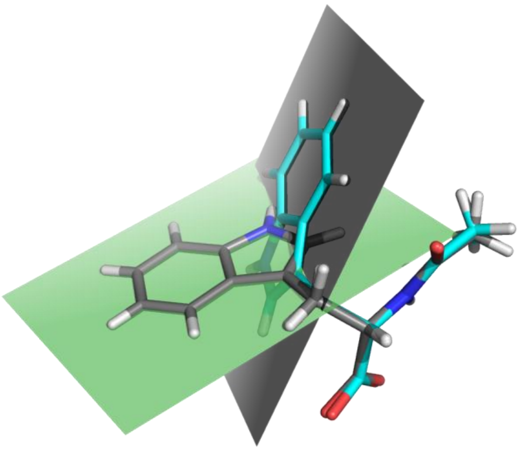

Figure S13. Amino acid conformations in **1**: overlap of the two non-equivalent tryptophan amino acids showing a mutual angle between indole sidechains of 53°.

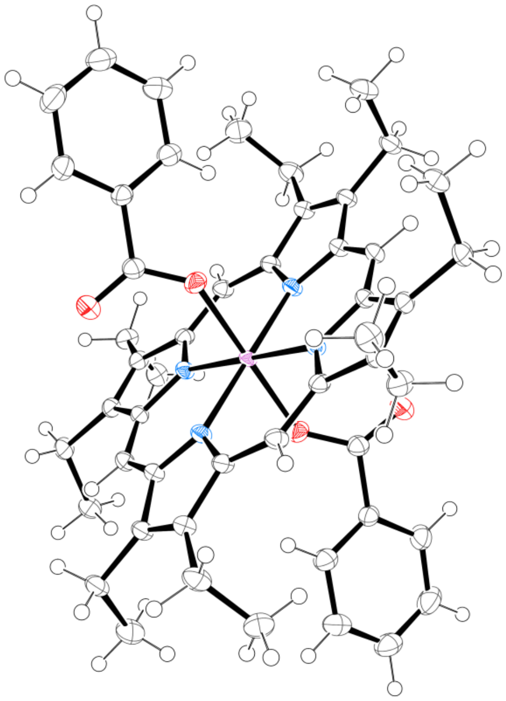

Figure S14. Ortep of the X-ray structure (50% probability) of **4**. Colour code: oxygen, red; nitrogen, blue; tin, purple. Half compound **4** was found in the crystallographic asymmetric unit, consistent with the presence of a crystallographic inversion center that matches the metal position.

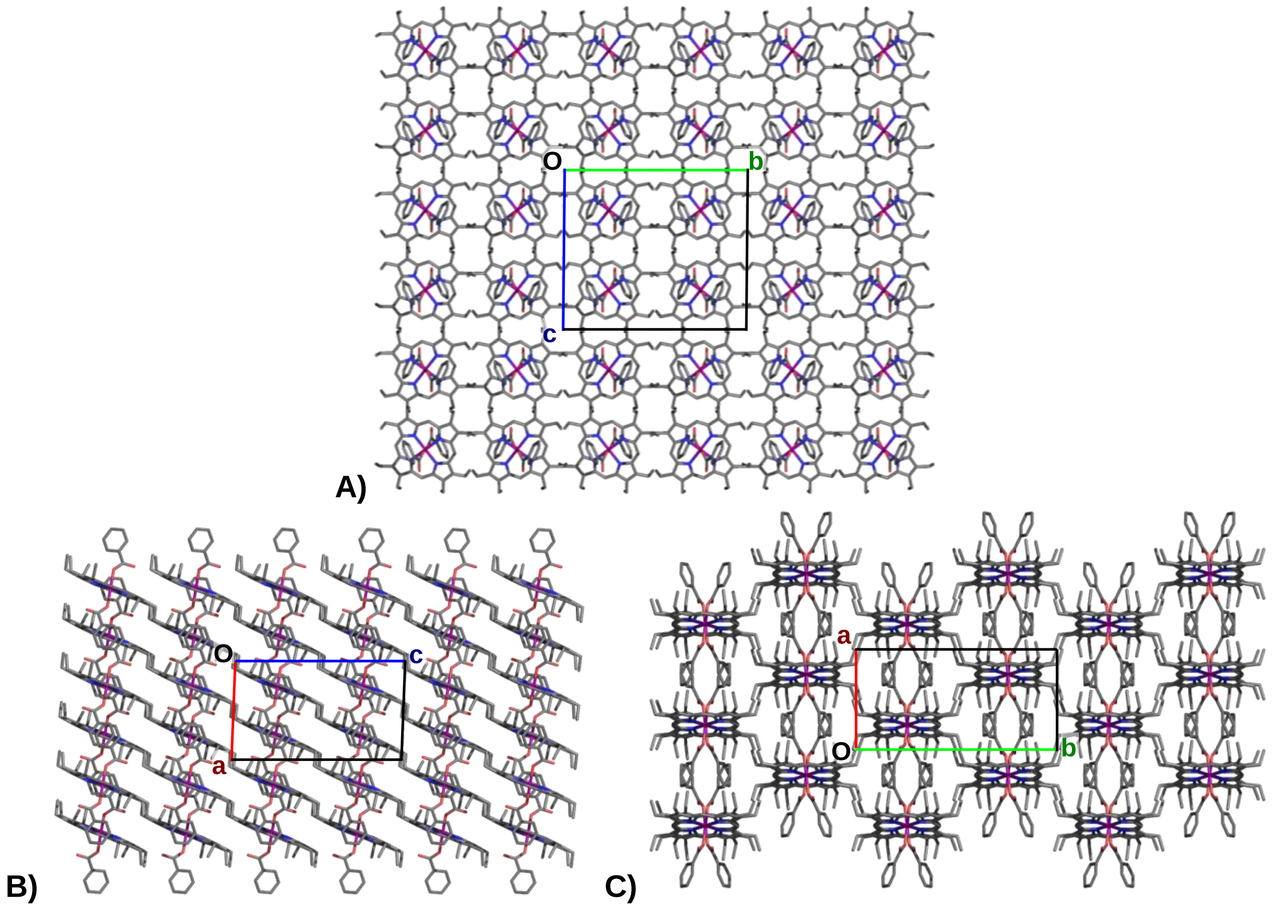

Figure S15. Crystal packing views of **4** along crystallographic A) *a*, B) *b* and C) *c* axis. Hydrogens omitted for clarity. The monoclinic crystal form of **4** shows a compact crystal packing, without solvent molecules inclusions. Hydrophobic contacts keep **4** moieties packed. The compound stack in pillars parallel to crystallographic *a* axis, where neighbor complexes are glued by $\pi\cdots\pi$ interactions between adjacent benzoic acid phenyl rings (perpendicular distance of adjacent ring centroids are 3.691(1) Å).

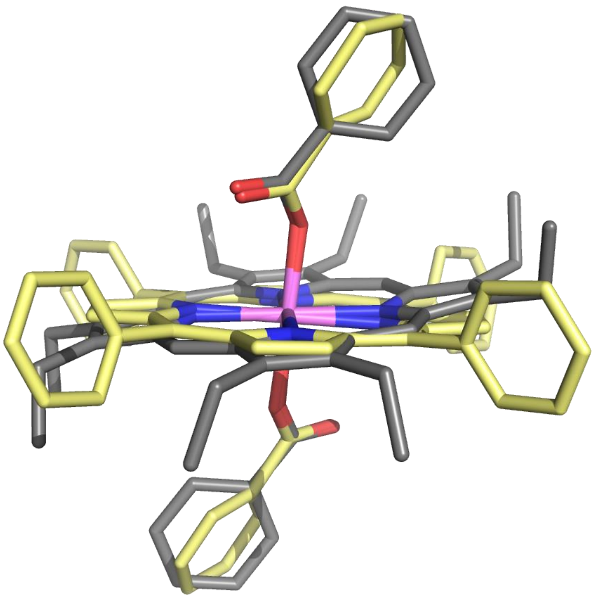

Figure S16. Overlap of the X-ray structure of **4** (Carbon skeleton in gray) and **3** (Carbon skeleton in yellow).[S7] The molecular structure of **4** is closely identical to that of **3**,[S7] and similar Sn(IV)-porphyrin carboxylate coordinated systems.[S7-S9]

S4. Electrochemical characterization

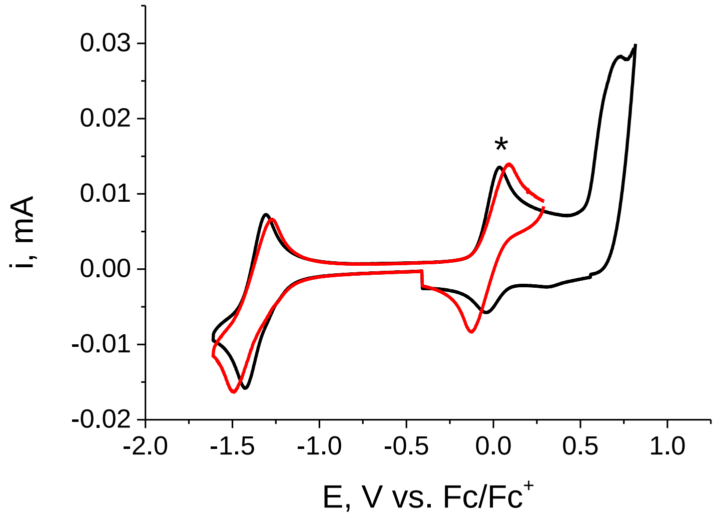

Figure S17. CV of **1** (black, full scan; red, cathodic scan only) in N_2-purged DCM (0.1 $TBAPF_6$) at room temperature, scan rate *v* = 100 mV/s, GC as WE, Pt as CE, SCE as reference, potential referred to the Fc/Fc^+ couple (*). The apparent irreversibility of the Fc/Fc^+ redox process in the full scan is attributable to electrode passivation after oxidation of tryptophan (see Figure S20).

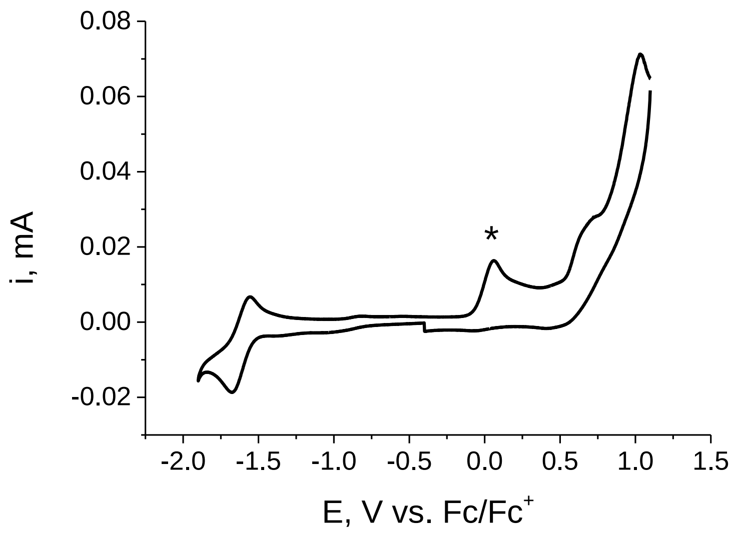

Figure S18. CV of **3** in N_2-purged DCM (0.1 $TBAPF_6$) at room temperature, scan rate v = 100 mV/s, GC as WE, Pt as CE, SCE as reference, potential referred to the Fc/Fc^+ couple (*). The apparent irreversibility of the Fc/Fc^+ redox process is attributable to electrode passivation after oxidation of tryptophan (see Figure S20).

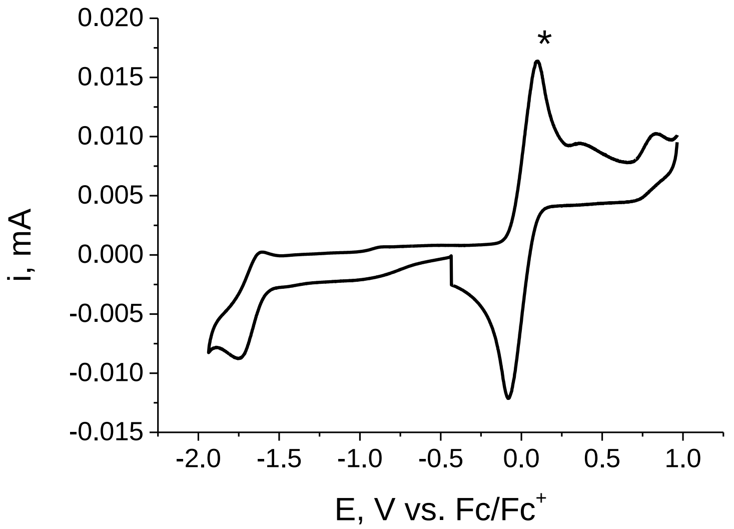

Figure S19. CV of **4** in N_2-purged DCM (0.1 $TBAPF_6$) at room temperature, scan rate v = 100 mV/s, GC as WE, Pt as CE, SCE as reference, potential referred to the Fc/Fc^+ couple (*).

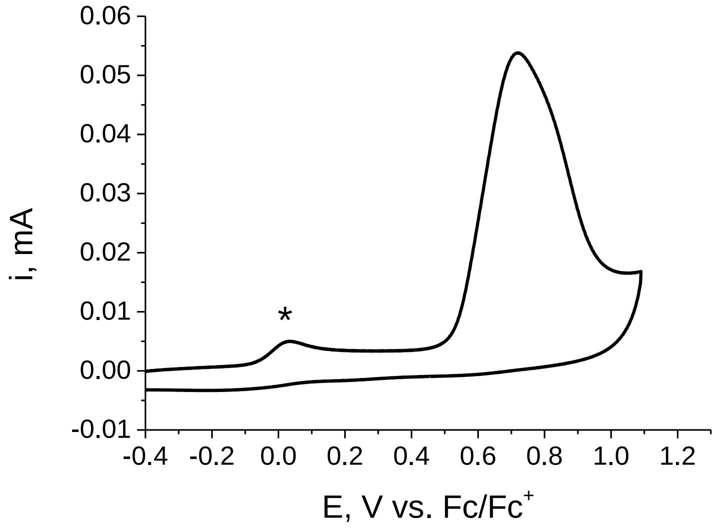

Figure S20. CV of N-acetyl-L-tryptophan in N_2-purged DCM (0.1 $TBAPF_6$) at room temperature, scan rate v = 100 mV/s, GC as WE, Pt as CE, SCE as reference, potential referred to the Fc/Fc^+ couple (*). The apparent irreversibility of the Fc/Fc^+ redox process is attributable to electrode passivation after oxidation of tryptophan.

S5. Photophysical characterization

S5.1 Photophysical characterization of conjugate 1 in CH_2Cl_2

The singlet excited state is quenched in **1** when compared to model compound **3**. An efficiency $\eta_{q,S_1}(\%)$ of 75% has been estimated for the SnTPP singlet quenching in conjugate **1** according to eq. S1, where I(**1**) and I(**3**) are the integrated emission intensities of the fluorescence of **1** and **3** (Figure S21), respectively.

$$\eta_{q,S_1}(\%) = \left(1 - \frac{I(\mathbf{1})}{I(\mathbf{3})}\right) \cdot 100 \qquad \text{(S1)}$$

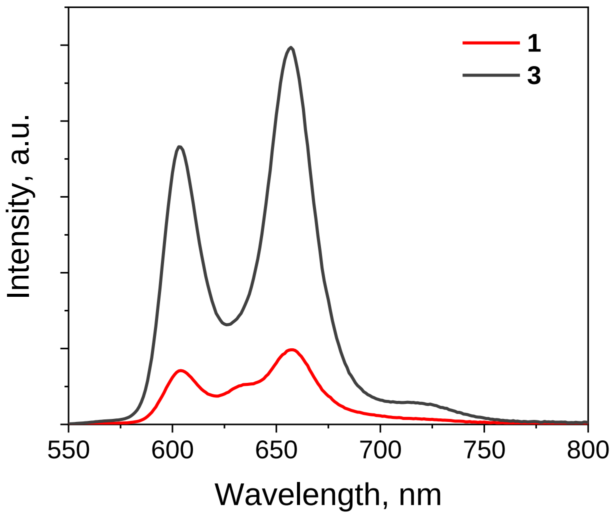

Figure S21. Fluorescence spectra of **1** and **3** in dichloromethane (excitation at 534 nm, optically matched solutions at the excitation wavelength).

An efficiency $\eta_T(\%)$ of 46% has been estimated for the SnTPP triplet formation in conjugate **1** according to eq. S2, where ΔOD(**1**) and ΔOD(**3**) are the maximum differential absorption of the

SnTPP triplet excited state measured at 480 nm (Figure S22) in conjugate **1** and model compound **3**, respectively, and 0.9 is the intersystem crossing yield of SnTPP in model **3**.[S10]

$$\eta_T(\%) = \left(0.9 \cdot \frac{\Delta OD(\mathbf{3})}{\Delta OD(\mathbf{1})}\right) \cdot 100 \qquad \text{(S2)}$$

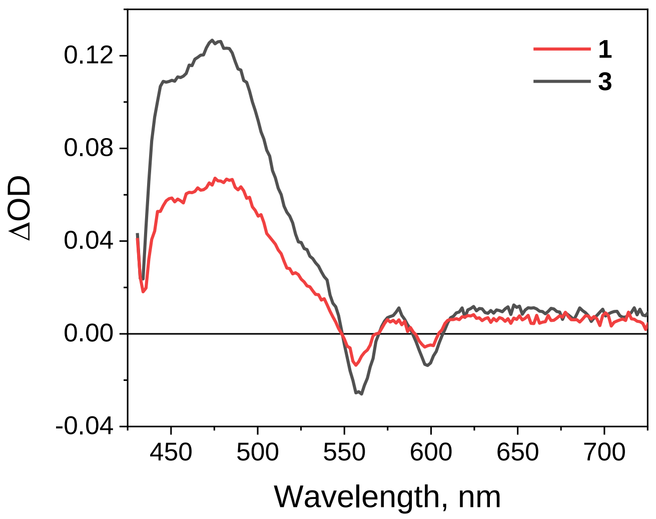

Figure S22. Transient absorption spectra of **1** and **3** in N_2-purged dichloromethane obtained by laser flash photolysis at 50 ns time-delay (excitation at 532 nm, optically matched solutions at the excitation wavelength).

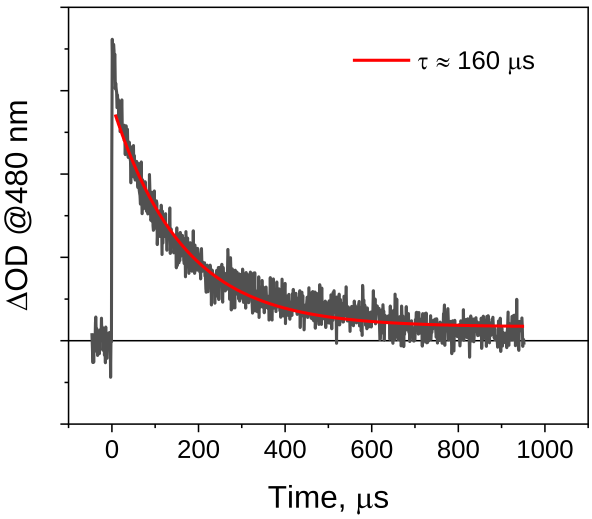

Figure S23. Kinetic trace at 480 nm of the triplet decay in **1** obtained by laser flash photolysis in N_2-purged dichloromethane (excitation at 532 nm, 2 mJ), the average lifetime of 160 μs has been estimated as the weighted mean value obtained from a bi-exponential fitting of the kinetic trace.

An energy level diagram (Figure S24) can be constructed to describe the photophysical behavior of conjugate **1** in dichloromethane using the energy of the singlet excited state (^{1}*SnTPP) obtained from the intersection of the normalized absorption/emission spectra of model compound **3**, the energy of the triplet excited state (^{3}*SnTPP) from phosphorescence data,[S10] and the energy of the charge transfer state from electrochemical data (see Table 1 of the main article).

The rate constant for the forward ET ($4.0 \cdot 10^{10}$ s^{-1}) has been taken from the reciprocal of the τ_1 obtained by UFS experiment (Figure 3 of the main text). The rate constants for triplet formation ($k_T = 1.5 \cdot 10^9$ s^{-1}), and charge recombination ($k_{CR} = 1.6 \cdot 10^9$ s^{-1}) have been calculated according to the combination of eqs. S3 and S4, where τ is the excited state lifetime in **1** (0.31 ns) measured by TC-SPC (see main text), $\eta_T = 0.46$ is the triplet formation yield in **1** (see above), and $k_S \sim 10^8$ s^{-1} is the rate constant for singlet decay to the ground state (comprising both fluorescence and internal conversion), assumed to be comparable to that of model compound **3**.[S10]

$$\begin{cases} \tau = \frac{1}{k_S + k_T + k_{CR}} & \text{(S3)} \\ k_{ISC} \cdot \tau = \eta_T & \text{(S4)} \end{cases}$$

The rate constants for triplet decay (~$6{\cdot}10^3$ s^{-1}) has been taken from the reciprocal of the excited state lifetime determined in Figure S23.

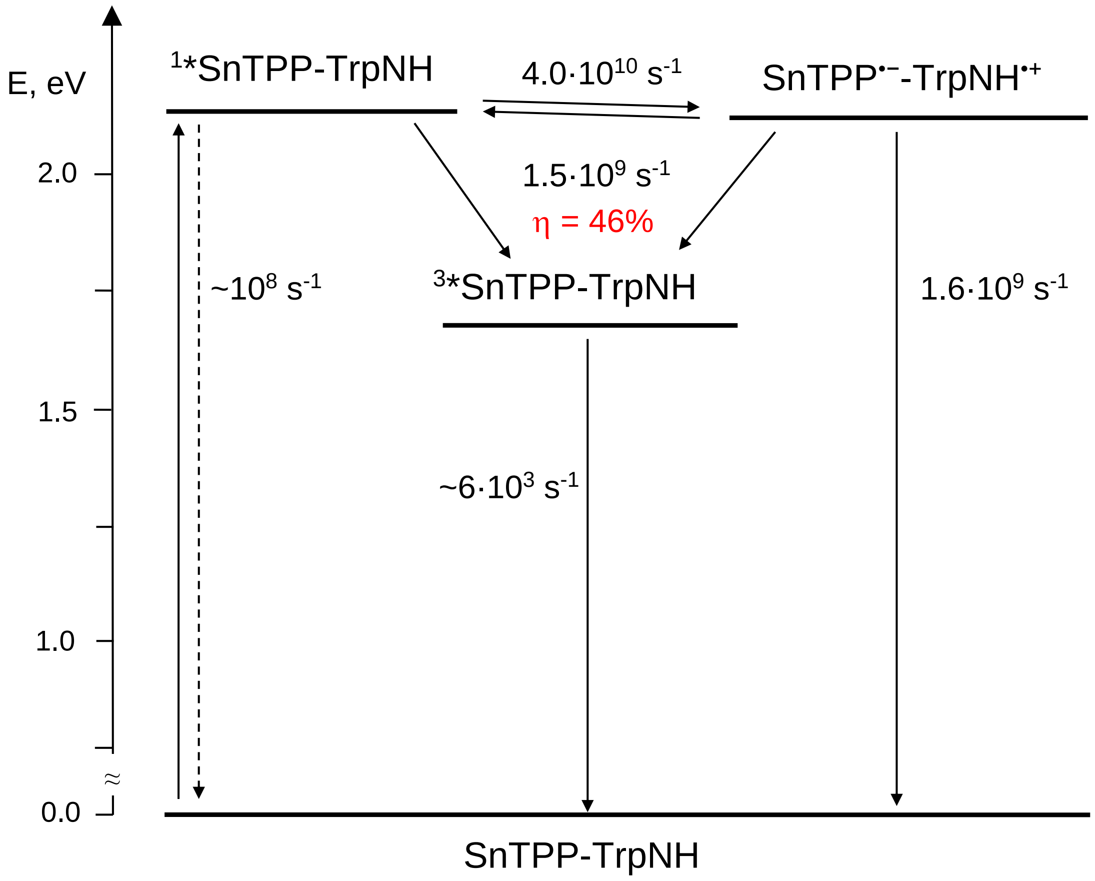

Figure S24. Energy level diagram with related kinetics describing the photophysical behavior of conjugate **1** in CH_2Cl_2.

S5.2 Photophysical characterization of conjugate 2 in CH_2Cl_2

Neither the singlet excited state nor the triplet excited state in **2** are quenched when compared to model compound **4**.

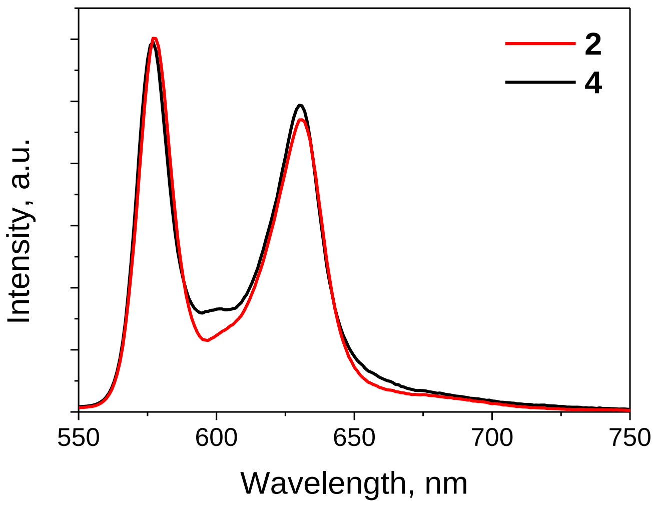

Figure S25. Fluorescence spectra of **2** and **4** in dichloromethane (excitation at 520 nm, optically matched solutions at the excitation wavelength).

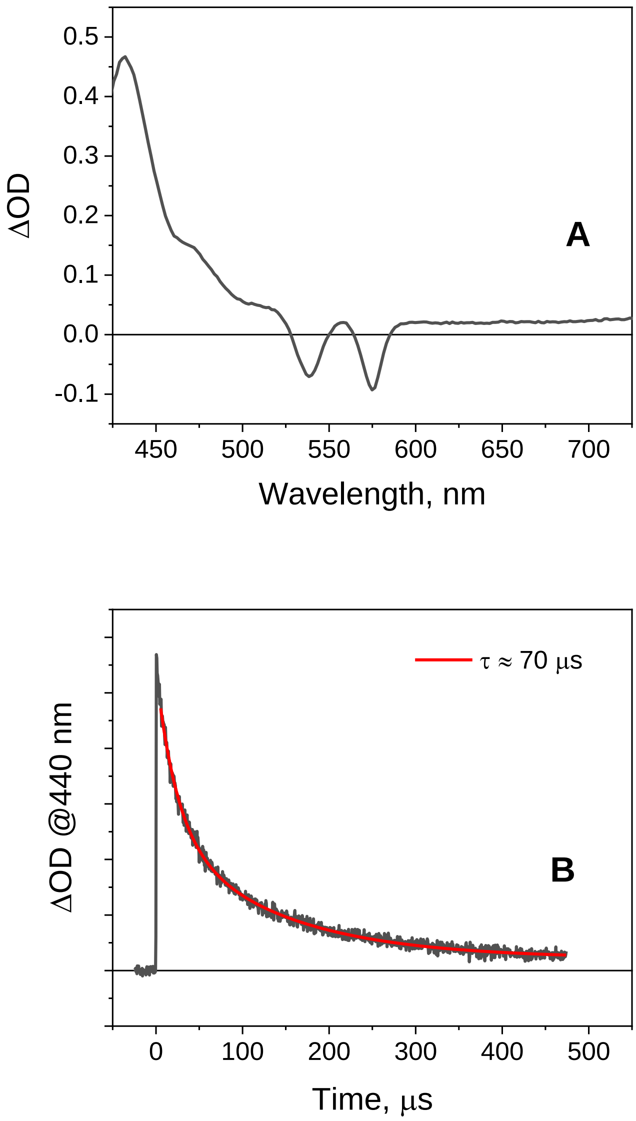

Figure S26. A) Transient absorption spectrum at 100 ns time delay and B) kinetic trace at 440 nm of the triplet decay in **2** obtained by laser flash photolysis in N_2-purged dichloromethane (excitation at 532 nm, 2 mJ), the average lifetime of 70 μs has been estimated as the weighted mean value obtained from a bi-exponential fitting of the kinetic trace.

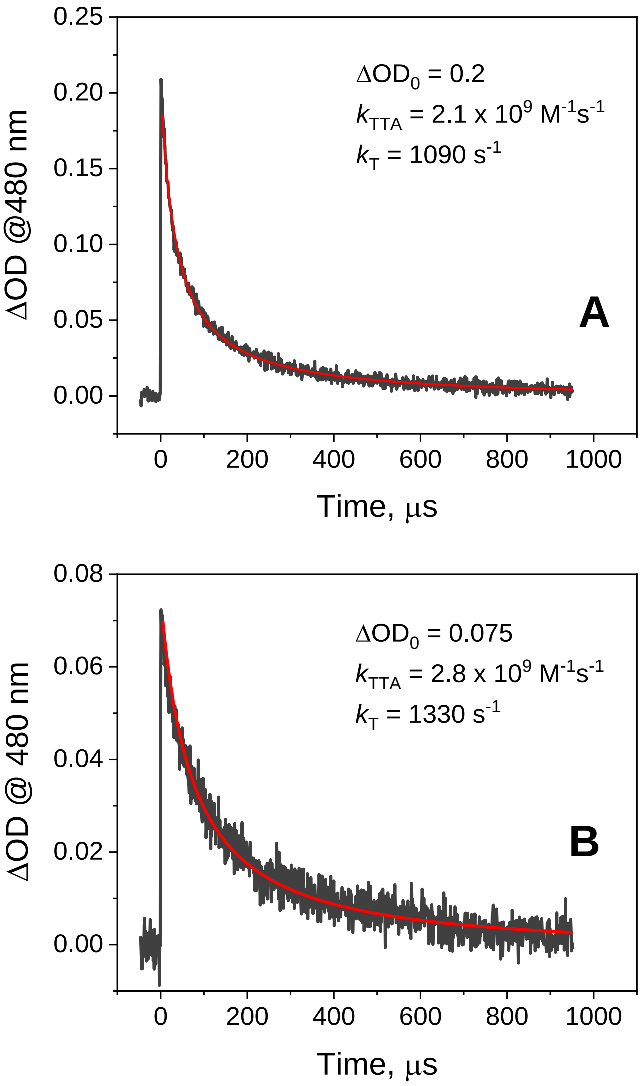

Figure S27. Kinetic traces at 480 nm of the triplet decay in A) **3** and B) **1** obtained by laser flash photolysis in N_2-purged dichloromethane (excitation at 532 nm, 2 mJ), the fitting has been performed according to eq S5 using a molar extinction coefficient of $\Delta\varepsilon$ = 22,000 $M^{-1}cm^{-1}$ at 480 nm[S11] and an optical pathlength of 0.74 cm (characteristic of our laser flash photolysis setup).[S12]

$$\Delta OD = \frac{\Delta\varepsilon \cdot l \cdot \Delta OD_0 e^{-k_T t}}{(\Delta\varepsilon \cdot l + (\Delta OD_0 \cdot k_{TTA}/k_T) - (\Delta OD_0 \cdot k_{TTA}/k_T) e^{-k_T t})} \quad \text{(S5)}$$

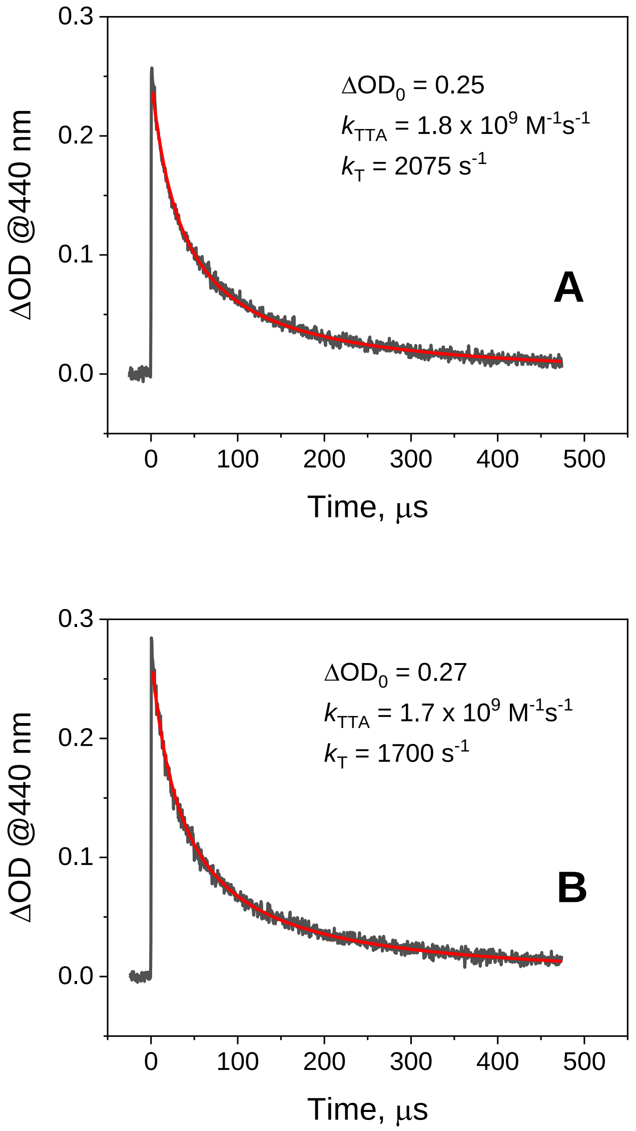

Figure S28. Kinetic traces at 480 nm of the triplet decay in A) **4** and B) **2** obtained by laser flash photolysis in N_2-purged dichloromethane (excitation at 532 nm, 2 mJ), the fitting has been performed according to eq S5 using a molar extinction coefficient of $\Delta\varepsilon$ = 24,000 $M^{-1}cm^{-1}$ at 440 nm (taken from a ZnOEP analogue)[S11] and an optical pathlength of 0.74 cm (characteristic of our laser flash photolysis setup).[S12]

An energy level diagram (Figure S29) can be constructed to describe the photophysical behavior of conjugate **2** in dichloromethane using the energy of the singlet excited state (^{1*}SnOEP) obtained from the intersection of the normalized absorption/emission spectra of model compound **4** and the energy of the triplet excited state (^{3*}SnOEP) from phosphorescence data.[S11]

The rate constant for the singlet decay ($1.1 \cdot 10^9$ s^{-1}) has been taken from the reciprocal of the τ obtained from TC-SPC (see main text). The rate constant for the singlet decay (~$1.4 \cdot 10^4$ s^{-1}) has been taken from the reciprocal of the lifetime determined in Figure S26B.

The photophysical behavior clearly resembles that of model compound **4** and related SnOEP.[S11,S13]

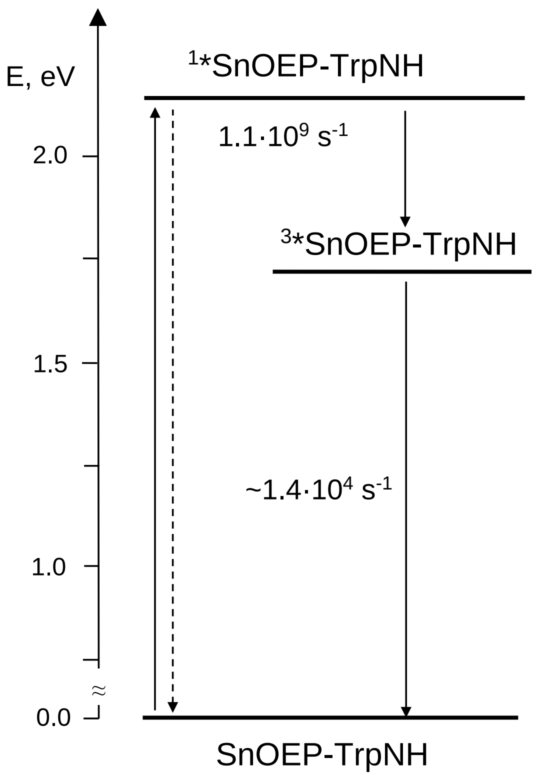

Figure S29. Energy level diagram with related kinetics describing the photophysical behavior of conjugate **2** in CH_2Cl_2.

S5.3 Determination of the association constant for H-bonding between N-acetyl-L-tryptophan and pyrrolidine in CH_2Cl_2

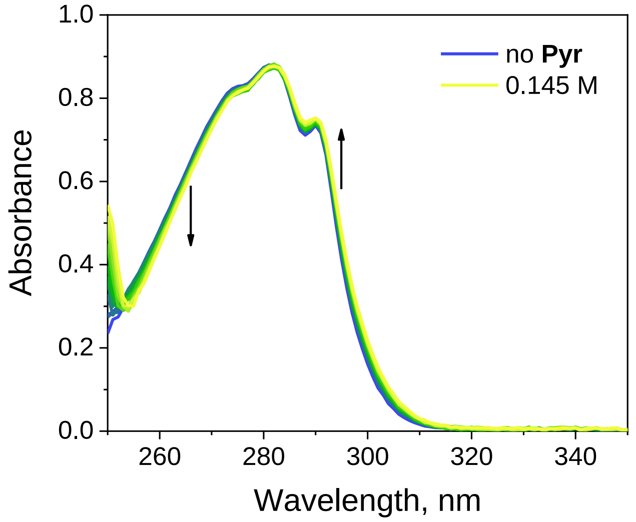

Figure S30. Absorption spectra of 0.15 mM N-acetyl-L-tryptophan in dichloromethane upon addition of 0-0.145 M pyrrolidine. Note that i) all spectra have been corrected considering the absorption from the added pyrrolidine, ii) the spectrum corresponding to the absence of pyrrolidine (no **Pyr**) is obtained with 0.15 mM pyrrolidine in order to deprotonate the carboxylic acid of the amino acid which is observed to affect the spectrum of pristine N-acetyl-L-tryptophan.

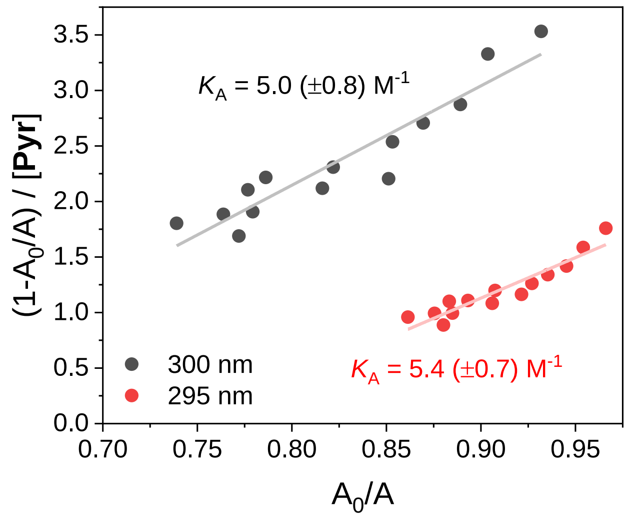

Figure S31. Treatment of the absorbance values at 295 and 300 nm using the procedure reported by Mataga and co-workers,[S14] the association constant K_A corresponds to the opposite of the intercept extracted from a linear fit of the experimental data.

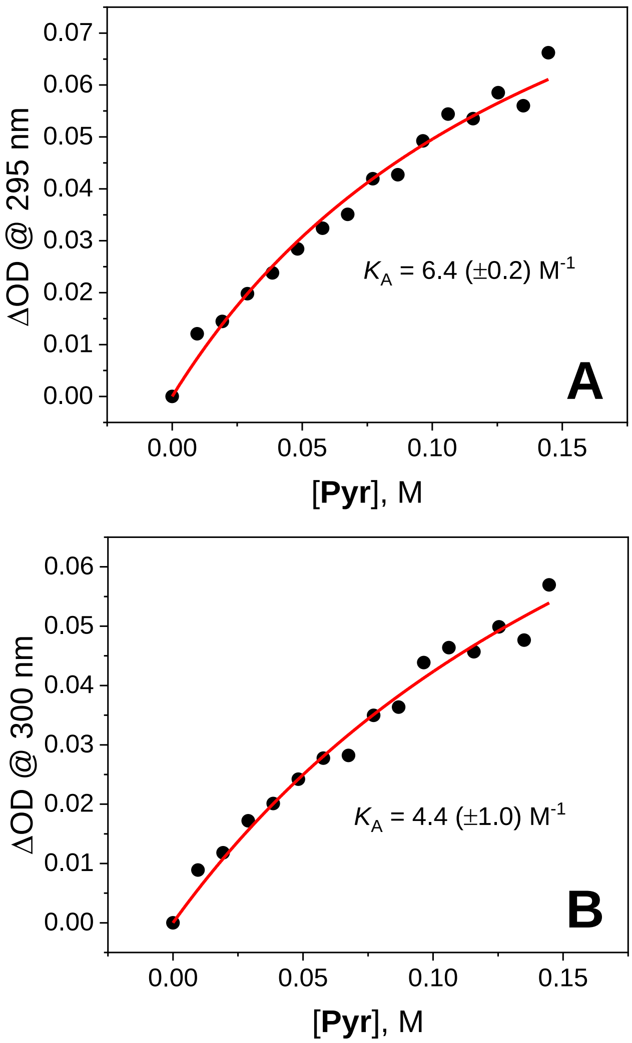

Figure S32. Plot of the ΔOD vs. pyrrolidine concentration at two different wavelengths and fitting according to the treatment by Hammarström and co-workers (eq S6).[S15]

$$[TrpNH \cdots Pyr] = \frac{[TrpNH]_0 + [Pyr]_0 + K_A^{-1}}{2} \mp \sqrt{\left(\frac{[TrpNH]_0 + [Pyr]_0 + K_A^{-1}}{2}\right)^2 - [TrpNH]_0[Pyr]_0} \quad \text{(S6)}$$

S5.4 Photophysics of conjugates 1 and 2 in CH_2Cl_2 in the presence of pyrrolidine

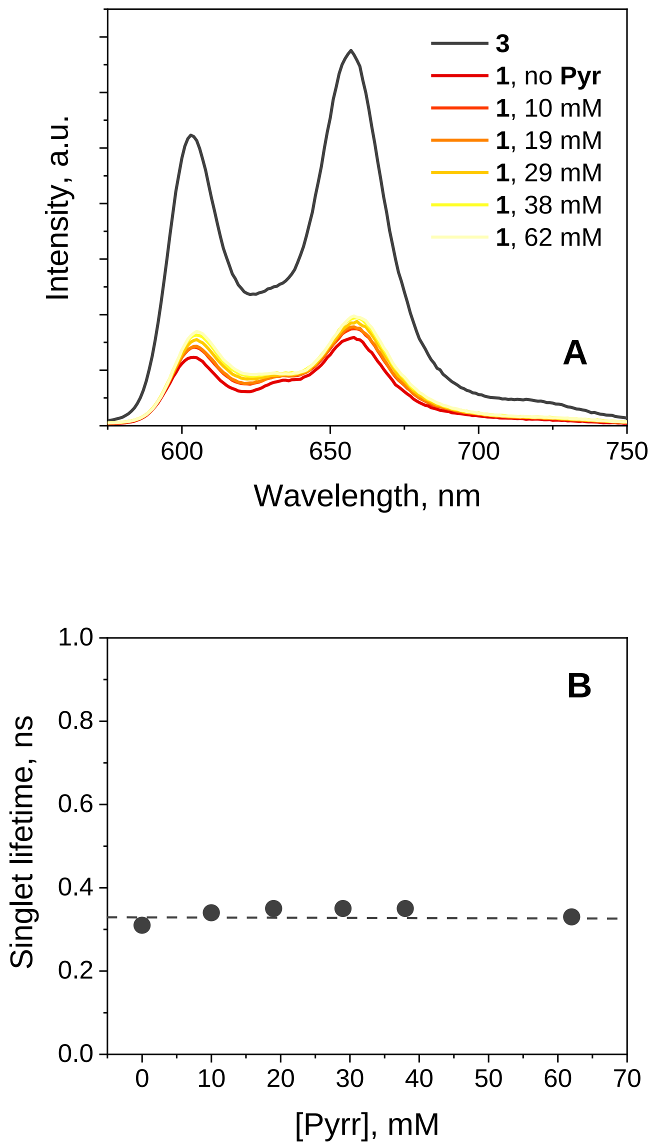

Figure S33. A) Fluorescence spectra (excitation at 534 nm) of **1** in dichloromethane in the presence of 0-0.062 M pyrrolidine and **3**, B) singlet excited state lifetime measured by TC-SPC (excitation at 600 nm, analysis at 660 nm).

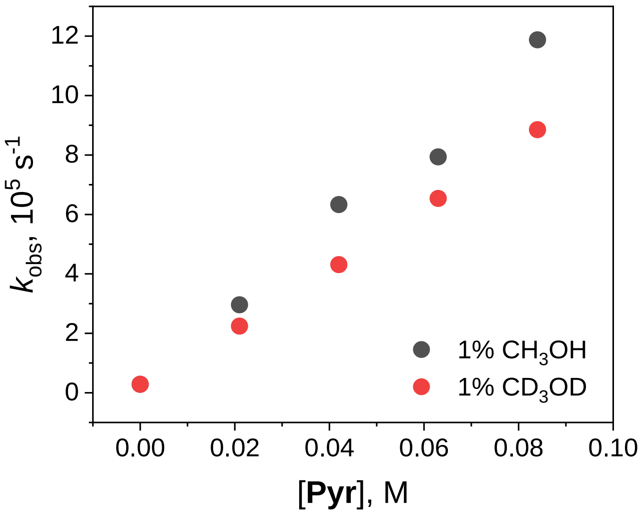

Figure S34. Rates of triplet excited state decay obtained from the kinetic traces at 480 nm measured by laser flash photolysis (excitation at 532 nm, 2 mJ) of **1** with 0-0.084 M pyrrolidine in N_2-purged CH_2Cl_2 in the presence of 1% CH_3OH and CD_3OD.

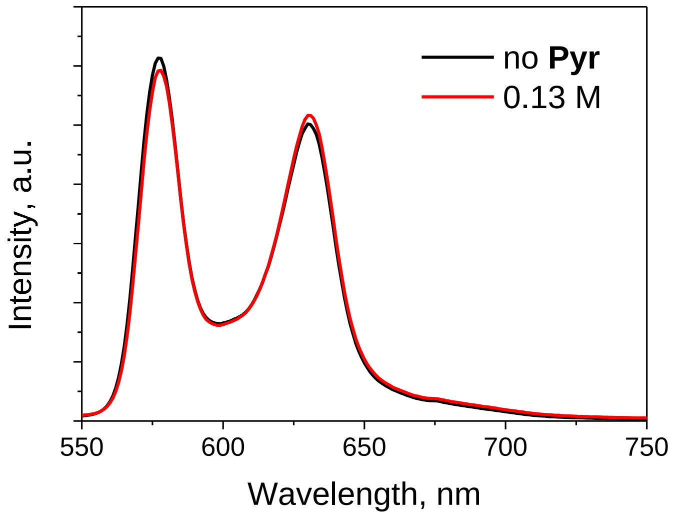

Figure S35. Fluorescence spectra of **4** in dichloromethane (excitation at 534 nm) in the presence and absence of 0.13 M pyrrolidine.

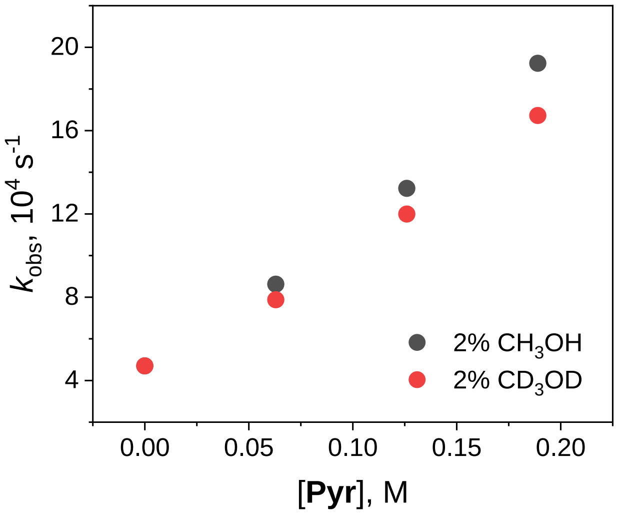

Figure S36. Rates of triplet excited state decay obtained from a single-exponential fitting of the kinetic traces at 430 nm measured by laser flash photolysis (excitation at 532 nm, 2 mJ) of **2** with 0-0.18 M pyrrolidine in N_2-purged CH_2Cl_2 in the presence of 2% CH_3OH and CD_3OD.

S6. Energy level diagrams

The energy levels of the radical pair state and the PT-only state in both **1** and **2** have been constructed as follows.

1) $SnP^{\bullet-}$-$TyrOH^{\bullet}\cdots{}^{+}HPyr$ level

For the sake of simplicity, the possible energy difference between the singlet and triplet radical pair states, which is related to the spin-spin exchange integral, has been neglected and the same energy has been thus assumed for both states.

The energy of the radical pair state has been estimated from eq S7:

$$E_{RP} = e\,[E\,(TrpNH^{\bullet+}/TrpNH) - E\,(SnP/SnP^{\bullet-})] + 0.059\ \text{eV} \cdot [pK_a\,(TrpNH^{\bullet+}) - pK_a\,({}^{+}HPyr)] \qquad (S7)$$

The potentials of the model compounds have been considered (Table 1 of the main text), a $pK_a \sim -1$ has been taken for the $TrpNH^{\bullet+}$ from the known pK_a value of $Ph_2NH^{\bullet+}$ in DMSO (a similar NH acid, $pK_a = 2.7$)[S16] and assuming an identical shift between the ground state pK_a and the pK_a of the oxidized species (ca 22 pK_a units as possibly estimated from $pK_a = 2.7$ for $Ph_2NH^{\bullet+}$, $pK_a = 25$ for Ph_2NH, and $pK_a = 21$ for indole),[S16] pK_a (${}^{+}HPyr$) = 11.1 has been taken from data in DMSO.[S17] An error of ±3 pK_a units has been assumed for the calculated ΔpK_a value which is related to the possible error arising from change in solvent (from DMSO to CH_2Cl_2).[S18]

2) SnP-TyrOH$^{-}\cdots{}^{+}$HPyr level

The energy of the PT-only state has been taken from eq S8

$$E_{PT\text{-}only} = 0.059\ \text{eV} \cdot [\text{p}K_a\ (\text{TrpNH}) - \text{p}K_a\ (^{+}\text{HPyr})] \qquad \text{(S8)}$$

The pK_a (TrpNH) = 21.0 in acetonitrile has been taken from available pK_a data of indole in DMSO,[S16] a pK_a ($^{+}$HPyr) = 11.1 has been taken from data in DMSO.[S17]

S7. References of the Supporting Information

[S1] A. Lausi, M. Polentarutti, S. Onesti, J. R. Plaisier, E. Busetto, G. Bais, L. Barba, A. Cassetta, G. Campi, D. Lamba, A. Pifferi, S. C. Mande, D. D. Sarma, S. M. Sharma, G. Paolucci, *Eur. Phys. J. Plus* **2015**, *130*, 1.

[S2] W. Kabsch, *Acta Cryst. D*, **2010**, *66*, 125.

[S3] G. M. Sheldrick, *Acta Cryst. A*, **2015**, *71*, 3.

[S4] P. Emsley, B. Lohkamp, W. G. Scott, K. Cowtan, *Acta Cryst. D*, **2010**, *66*, 486.

[S5] L. Farrugia, *J. Appl. Cryst.*, **2012**, *45*, 849.

[S6] L. Schrodinger, The PyMOL Molecular Graphics System, Schrodinger, LLC, **2015** http://www.pymol.org

[S7] a) G. Smith, D. P. Arnold, C. H. L. Kennard, T. C. W. Mak, *Polyhedron* **1991**, 10, 509. b) S. H. Kim, H. Kim, K.K. Kim, H. J. Kim, *J. Porph. Phthal.*, **2009**, *13*, 805.

[S8] M. Natali, A. Amati, N. Demitri, E. Iengo, *Chem. Commun.*, **2018**, *54*, 6148.

[S9] P. Cavigli, G. Balducci, E. Zangrando, N. Demitri, A. Amati, M. T. Indelli, E. Iengo, *Inorg. Chim. Acta*, **2016**,*439*, 61.

[S10] M. Casanova, E. Zangrando, E. Iengo, E. Alessio, M. T. Indelli, F. Scandola, M. Orlandi, *Inorg. Chem.*, **2008**, *47*, 10407.

[S11] K. Kalyanasundaram, *Photochemistry of Polypyridine and Porphyrin Complexes*, Academic Press, **1992**.

[S12] M. Natali, M. Orlandi, S. Berardi, S. Campagna, M. Bonchio, A. Sartorel, F. Scandola, *Inorg. Chem.*, **2012**, *51*, 7324.

[S13] A. Amati, P. Cavigli, N. Demitri, M. Natali, M. T. Indelli, E. Iengo, *Inorg. Chem.*, **2019**, *58*, 4399

[S14] H. Miyasaka, A. Tabata, S. Ojima, N. Ikeda, N. Mataga, *J. Phys. Chem.*, **1993**, *97*, 8222.

[S15] J. Petersson, L. Hammarström, *J. Phys. Chem. B*, **2015**, *119*, 7531.

[S16] J. J. Warren, T. A. Tronic, J. M. Mayer, *Chem. Rev.*, **2010**, *110*, 6961.

[S17] R. Crampton, I. A. Robotham, *J. Chem. Res. (S)*, **1997**, 22.

[S18] A. Kütt, S. Selberg, I. Kaljurand, S. Tshepelevitsh, A. Heering, A. Darnell, K. Kaupmess, M. Piirsalu, I. Leito, *Tetrahedron Lett.*, **2018**, *59*, 3738.
